# Mouse T_H_2 cell extracellular vesicles promote eosinophil survival through the surface cytokine cargo IL-3

**DOI:** 10.1016/j.jaci.2025.05.027

**Published:** 2025-06-10

**Authors:** Kaitlyn E. Bunn, Brenna G. Giese-Byrne, Alexander M. Blatt, Dawn C. Newcomb, Heather H. Pua

**Affiliations:** a Department of Pathology, Microbiology, and Immunology, Vanderbilt University Medical Center, Nashville; b Department of Medicine, Vanderbilt University Medical Center, Nashville; c Vanderbilt Institute for Infection, Immunology, and Inflammation, Vanderbilt University Medical Center, Nashville; d Vanderbilt Center for Immunobiology, Vanderbilt University Medical Center, Nashville; e Vanderbilt Center for Extracellular Vesicle Research, Vanderbilt University, Nashville.

**Keywords:** Extracellular vesicles, asthma, T_H_2 cells, eosinophils, IL-3, lung, allergic, type 2 inflammation

## Abstract

**Background:**

Extracellular vesicles (EVs) mediate intercellular communication during immune responses. EVs are abundant in respiratory biofluids, and the composition of EVs in the lung changes during inflammation.

**Objective:**

We sought to quantify the contribution of T cells to airway EVs in eosinophilic lung inflammation and ascertain their function during a type 2 inflammatory response.

**Methods:**

Genetic membrane tagging was combined with single vesicle flow cytometry to quantify T-cell EVs in the airways of mice challenged with ovalbumin or house dust mite. EVs were purified from mouse T_H_2 cell cultures, and their functions on eosinophils were assessed by flow cytometry and RNA sequencing. T_H_2 cell EVs were instilled into the lungs of mice to determine effects on lung eosinophilia. Finally, the function of an EV protein cargo was tested using inhibitors and blocking antibodies.

**Results:**

T-cell EVs are increased in the airways of mice after ovalbumin- or house dust mite–induced inflammation. EVs secreted by T_H_2 cells inhibit apoptosis and induce activating pathways in eosinophils *in vitro*. This effect depends on restimulation through the T-cell receptor. T_H_2 cell EVs prolong eosinophilia *in vivo* during acute eosinophilic inflammation. T_H_2 cell EVs carry the cytokine IL-3 as a surface cargo, which inhibits apoptosis by activating JAK1/2-dependent pro-survival programs in eosinophils.

**Conclusions:**

T_H_2 cell EVs promote eosinophil survival through the EV cargo IL-3, supporting a role for EVs as vehicles of cytokine-based communication in lung inflammation.

## Capsule summary

This study demonstrates that T-cell EVs promote the survival of eosinophils through the cytokine cargo IL-3, offering new insights into proinflammatory signaling in eosinophilic airway inflammation.

## Graphical Abstract

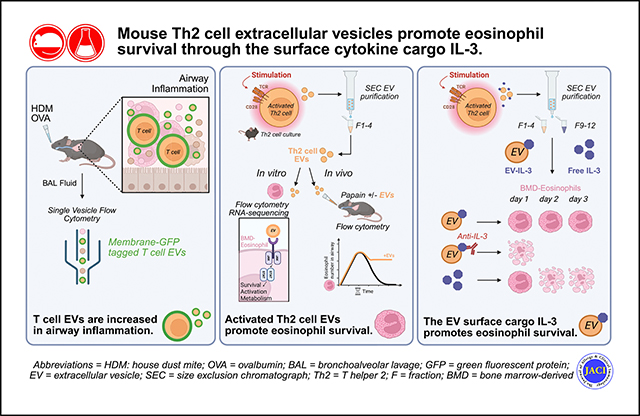

Asthma is a multicellular inflammatory disease of the airway driven by complex communication networks. A common endotype of asthma is defined by type 2 airway inflammation, with the recruitment and activation of T_H_2 cells, eosinophils, mast cells, and IgE-producing B cells.^1^ These immune cells produce cytokines, chemokines, and bioactive lipids that serve as key drivers of disease.^2,3^ Extracellular vesicles (EVs) have recently emerged as a new class of secreted factors produced by immune cells that participate in cell-to-cell communication.^4^ Yet their functions in type 2 tissue inflammation are poorly understood.

EVs are small, lipid bilayer-delimited particles secreted by cells that carry bioactive proteins, nucleic acids, and lipids.^5^ Cargos on the surfaces of EVs can signal through interactions with receptors on the surfaces of target cells, whereas cargos carried within the lumen of EVs can be delivered by fusion of the EV and target cell membranes.^6^ Respiratory biofluids contain abundant EVs, with concentrations ranging from 10^8^ to 10^10^ EVs/mL.^7–10^ Studies in mice and humans have shown that EVs from epithelial cells, endothelial cells, and immune cells are present in respiratory fluids and that their composition changes with inflammation.^11,12^ In particular, EVs originating from immune cells are increased in respiratory biofluids collected from patients with chronic rhinosinusitis, from patients with asthma, and in mouse models of type 2 airway inflammation.^13–15^ Immune cell EVs not only mark biofluids during inflammation, but also participate in type 2 immune responses through antigen presentation to allergen-specific T cells and the promotion of T_H_2 cell polarization.^16–18^

Similar to other immune cells, T cells secrete EVs. These EVs have been shown to carry cargos involved in T-cell immune functions, including cytokines, chemokines, microRNAs, tRNA fragments, DNA, integrins, and the T-cell receptor (TCR) complex.^19–24^ T-cell EVs can modulate the function of other immune cells through delivery of microRNAs, cytokines, and death-inducing ligands to target cells.^25–28^ In this study, we investigated an EV-mediated T cell–eosinophil communication axis as part of the type 2 inflammatory response in the airway.

## METHODS

### Animals

All animal studies were performed after obtaining approval from the Vanderbilt Institutional Animal Care and Use Committee.

### Mouse airway inflammation models

Ovalbumin (OVA) mice were sensitized intraperitoneally with 50 μg OVA and alum; challenged with 50 μg OVA by oropharyngeal aspiration on days 7, 8, and 9 following sensitization; and euthanized on day 10. House dust mite (HDM) mice were challenged with 40 μg HDM extract by oropharyngeal aspiration 3 times per week for 3 weeks and then euthanized the day following the last challenge. Papain mice were challenged with 3.5 μg papain by oropharyngeal aspiration and euthanized 24 to 72 hours following challenge. Bronchoalveolar lavage fluid (BALF) and lungs were collected, processed, and analyzed by flow cytometry. In a subset of mice, airway hyperresponsiveness studies were performed.

### Purification of EVs from primary T_H_2 cell cultures

CD4^+^ T cells were isolated from mouse spleen and lymph node single-cell suspensions by positive bead selection. Isolated cells were polarized to T_H_2 cells by stimulation with anti-CD3, anti-CD28, IL-4, and anti-IFN-γ for 3 days. On day 3, cells were removed from anti-CD3/anti-CD28 stimulation and rested in media with IL-4 and IL-2. On day 5, cell culture media was centrifuged at 250*g* for 10 minutes to pellet cells. The supernatant was collected and centrifuged at 2000*g* for 30 minutes to pellet large cellular debris and was used for purification of rested T_H_2 cell EVs. The cells were then stimulated with anti-CD3, anti-CD28, and IL-2 for 6 hours, and media were subjected to 250*g* and 2000*g* differential centrifugation and were used for isolation of activated T_H_2 cell EVs. EVs were isolated from cell culture supernatant by size exclusion chromatography (SEC) using qEV columns (Izon Science, Christchurch, New Zealand) per manufacturer instructions. Fractions were concentrated using 100 kDa ultrafiltration. EVs were characterized by nanoparticle tracking analysis, transmission electron microscopy, and single-vesicle flow cytometry.

### *In vitro* eosinophil cultures

Eosinophils were differentiated from mouse bone marrow by culturing bone marrow cells with FLT3 ligand and stem cell factor on days 0 to 3 and IL-5 on days 4 to 12. Eosinophils were characterized by microscopy, flow cytometry, and functional studies. On culture day 12, mature eosinophils were incubated with 5 × 10^9^ EVs/mL of rested and activated T_H_2 cell EVs for 6 or 24 hours, and viability was assessed by membrane permeability, caspase 3 activity, and annexin V binding. For RNA sequencing studies, eosinophils were treated with activated T_H_2 cell EVs or parallelly processed unconditioned media (GSE253180; https://www.ncbi.nlm.nih.gov/geo/). For ruxolitinib studies, eosinophils were treated with ruxolitinib or dimethyl sulfoxide vehicle control for 30 minutes at 37°C before the addition of EVs. For IL-3 blocking studies, EVs were incubated with anti-IL-3 or isotype control antibody for 30 minutes at 37°C before eosinophil treatment.

### Statistical analysis

Data were expressed as mean ± SEM or as median and interquartile range depending on the normality of the data. Statistical significance was determined through tests denoted in the figure legends, performed by GraphPad Prism 10 software (GraphPad Software, Boston, Mass). *P* < .05 was considered statistically significant.

Additional methodologic details for T-cell and eosinophil culture, EV purification, EV characterization (nanoparticle tracking analysis, transmission electron microscopy, single-vesicle flow cytometry), mouse allergic airway models including airway hyperresponsiveness, *in vivo* administration of EVs, cell viability assays, IL-3 detection and blocking, and RNA sequencing are provided in this article’s Methods section in the Online Repository available at www.jacionline.org.

## RESULTS

### T-cell EVs are increased in type 2 lung inflammation

**FIG 1.**
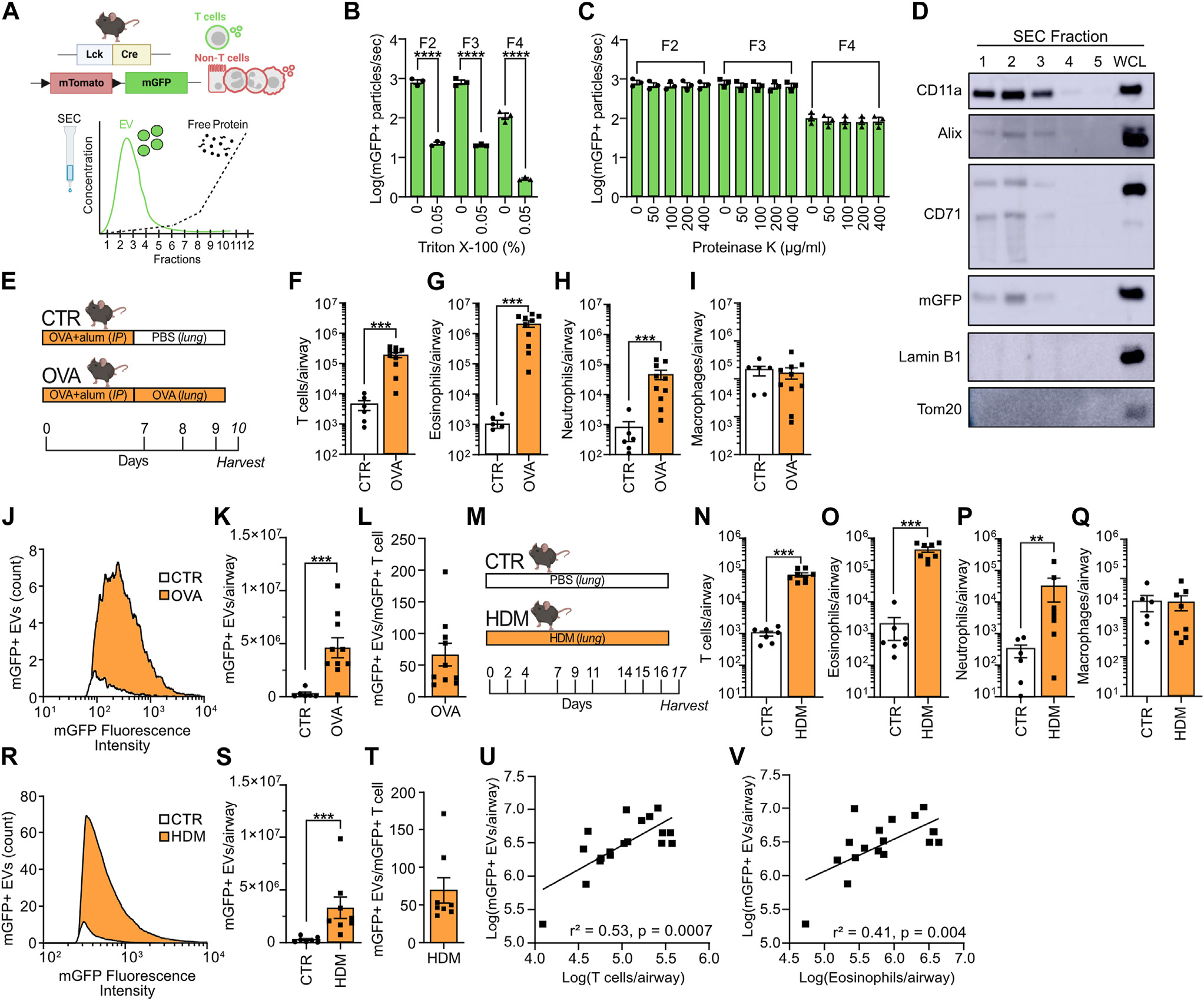
T-cell EVs are increased in type 2 lung inflammation. **(A)** Graphical depiction of the *Lck-Cre mTmG* mouse model and SEC fractionation of T-cell culture media. **(B** and **C)** Quantification of mGFP^+^ particles detected per second in *Lck-Cre mTmG* T-cell culture media by single-vesicle flow cytometry following *(B)* Triton X-100 treatment and *(C)* proteinase K treatment. n = 3, 2-way ANOVA with Sidak test for multiple comparisons. **(D)** Western blot of lysates obtained by SEC fractionation of *Lck-Cre mTmG* T_H_2 cell culture supernatant at 6 hours of culture. Equal loading volume between fractions. **(E** and **M)** Graphical depiction of *(E)* OVA and *(M)* HDM airway inflammation models. **(F-I** and **N-Q)** Quantification of the number of *(F* and *N)* T cells, *(G* and *O)* eosinophils, *(H* and *P)* neutrophils, and *(I* and *Q)* macrophages in the airways of *(F-I)* OVA and *(N-Q)* HDM mice following induction of airway inflammation. **(J** and **R)** Averaged histograms depicting the number of EVs corresponding to mGFP fluorescence intensity in *(J)* OVA and *(R)* HDM mice detected by single-vesicle flow cytometry in BALF. **(K** and **S)** Quantification of mGFP^+^ particles in the airways of *(K)* OVA and *(S)* HDM mice. **(L** and **T)** Quantification of the number of mGFP^+^ particles per mGFP^+^ T cell in the airways of *(L)* OVA and *(T)* HDM mice. **(U** and **V)** Correlation between mGFP^+^ particles and *(U)* T cells or *(V)* eosinophils in the airways of mice with induced type 2 airway inflammation (OVA and HDM experiments combined). *(F-L)* n = 6 CTR, n = 10 OVA, 2 independent experiments, 2-tailed Mann-Whitney test. *(N-T)* n = 7 CTR, n = 8 HDM, 2 independent experiments, 2-tailed Mann-Whitney test. *(U* and *V)* n = 10 OVA, 8 HDM, 4 independent experiments, simple linear regression. By flow cytometry, T cells were defined as TCRβ^+^ and CD4^+^ or CD8^+^, eosinophils were defined as Siglec F^+^ CD11b^+^ SSC^high^, neutrophils were defined as Ly6G^+^ CD11b^+^, and macrophages were defined as F4/80^+^ CD11c^+^. A full gating scheme can be found in Fig E1, B. All error bars represent SEM. **P < .01, ***P < .001, ****P < .0001. *CTR,* Control (PBS challenged); *WCL,* whole-cell lysate.

To test whether T-cell EVs are produced in mouse models of type 2 airway inflammation, we used a strategy previously developed in the laboratory to trace the cellular origins of EVs *in vivo*.^15^ We crossed *mTmG* mice^29^ with *Lck-Cre* mice^30^ to switch the expression of membrane-targeted Tomato for membrane-targeted green fluorescent protein (mGFP) in T cells expressing Cre recombinase (Fig 1, A). As expected, mGFP uniquely labeled T cells in these mice (Fig E1, A, in the Online Repository at www.jacionline.org). By single-vesicle flow cytometry, we detected mGFP^+^ EVs from *in vitro* cultured *Lck-Cre mTmG* CD4^+^ T cells, and these mGFP^+^ EVs could be degraded by the detergent Triton X-100, but not proteinase K (Fig 1, B and C). GFP in EVs purified by SEC also co-fractionated with the EV marker ALIX and the T-cell membrane markers CD11a and CD71, without contaminating subcellular membranes (Lamin B1—nucleus; Tom20—mitochondria) (Fig 1, D).

Having confirmed that EVs secreted by *Lck-Cre mTmG* T cells are labeled with mGFP, we next induced airway inflammation in *Lck-Cre mTmG* mice by OVA or HDM sensitization and challenge. Cellular flow cytometry on BALF showed a significant increase in airway T cells and eosinophils in OVA- and HDM-challenged mice, indicating the development of type 2 inflammation (Fig 1, E–I and M–Q; Fig E1, B), with 50% of airway T cells labeled with mGFP (Fig E1, C and D). To quantify mGFP-labeled EVs in the airway, we performed single-vesicle flow cytometry on BALF. We detected a mean of 4.62 × 10^6^ and 3.31 × 10^6^ mGFP^+^ EVs/airway in OVA- and HDM-challenged mice, respectively, which was significantly higher than in control mice (Fig 1, J and K and R and S). The linear range of detection of mGFP^+^ EVs from BALF was established by serial dilution studies, and the limit of detection was defined by running filtered PBS blanks (Fig E1, E). Loss of mGFP after Triton X-100 treatment supported that mGFP in BALF of *Lck-Cre mTmG* mice was carried by membrane-bound EVs (Fig E1, F). When the number of T-cell EVs was compared relative to the number of T cells in the airway, there were on average 67 and 69 mGFP^+^ EVs/mGFP^+^ T cell in OVA- and HDM-challenged mice, respectively (Fig 1, L and T). The number of mGFP^+^ T-cell EVs in the BALF of challenged mice positively correlated with both the number of T cells and the number of eosinophils in the BALF (Fig 1, U and V). There was also a positive correlation between T-cell EV number and airway resistance (Fig E1, G and H). Together, these data show that T cells secrete EVs in the airway during active type 2 lung inflammation.

### T_H_2 cell EVs promote eosinophil survival *in vitro*

**FIG 2.**
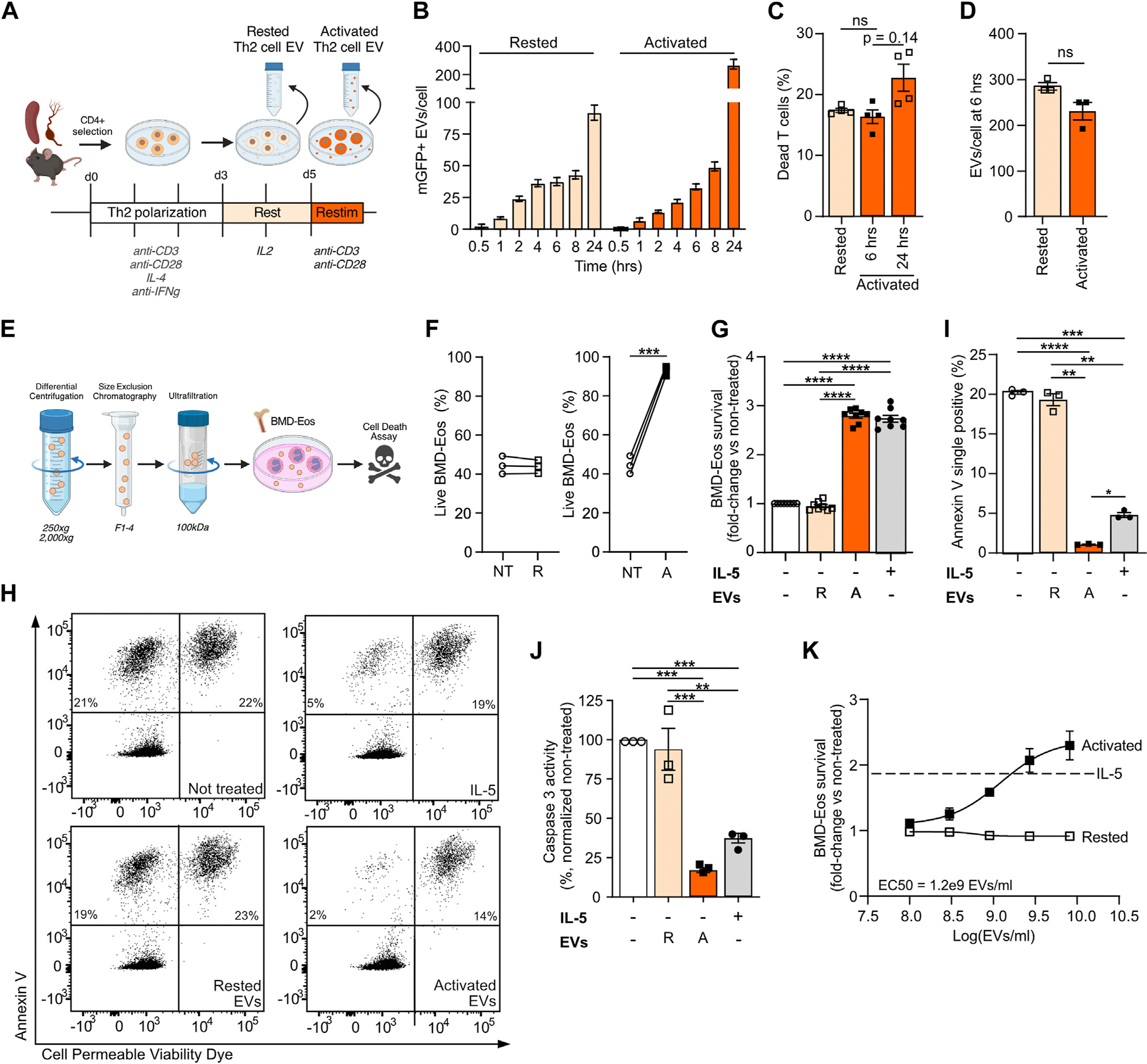
T_H_2 cell EVs promote eosinophil survival *in vitro*. **(A)** Graphical depiction of T_H_2 polarization of primary mouse T cells. **(B)** Quantification of EV production over time by rested and activated T_H_2 cells by single-vesicle flow cytometry. n = 3 for 0.5 to 8 hours, n = 2 for 24 hours. **(C)** Quantification of T-cell death at 6 and 24 hours following activation. n = 4, 1-way ANOVA (vs rested) with Bonferroni correction for multiple testing. **(D)** Quantification of EV production by rested and activated T_H_2 cells at 6 hours of culture by nanoparticle tracking analysis. n = 3, 2-tailed *t* test. **(E)** Graphical depiction of EV purification from T_H_2 cell culture media and treatment of bone marrow derived eosinophils. **(F)** Representative experiment showing quantification of percent survival of rested *(left)* and activated *(right)* T_H_2 cell EV–treated eosinophils by dye permeability assay at 24 hours post-treatment with 5 × 10^9^ EVs/mL. n = 3, 2-tailed paired *t* test, representative of 3 independent experiments. **(G)** Quantification of eosinophil survival by dye permeability assay as fold change over the nontreated condition at 24 hours post-treatment with 5 × 10^9^ T_H_2 cell EVs/mL; representative experiment showing % survival is shown in *(F)*. n = 9, 3 independent experiments, 1-way ANOVA with Tukey multiple comparisons test. **(H)** Representative flow cytometry plots showing % annexin V and permeability dye-stained cells of eosinophils following 24 hours of treatment with no cytokine or EVs, 10 ng/mL IL-5, or 5 × 10^9^ rested or activated T_H_2 cell EVs/mL. **(I)** Quantification of annexin V^+^ permeability dye^−^ eosinophils following 6 hours of treatment with 5 × 10^9^ T_H_2 cell EVs/mL. **(J)** Quantification of caspase-3 activity in eosinophils following 6 hours of treatment with 5 × 10^9^ T_H_2 cell EVs/mL. For *(I* and *J)*, n = 3, 1-way ANOVA with Tukey multiple comparisons test. **(K)** Quantification of eosinophil survival following 24 hours of treatment with increasing doses of T_H_2 cell EVs, n = 3, log[agonist] versus response with variable response (4 parameters). All error bars represent SEM, **P < .01, ***P < 0.001, ****P < .0001. *A,* Activated; *BMD-eos,* bone marrow derived eosinophils; *ns,* not significant; *NT,* no treatment; *R,* rested.

Given the critical role of T cells in driving type 2 airway inflammation, we next investigated EV secretion from *in vitro* polarized T_H_2 cells that were either re-stimulated through the TCR (activated) or not (rested) following polarization (Fig 2, A). To avoid contamination from serum-derived EVs, T cells were polarized in serum-free media with T_H_2 cell polarization confirmed by cytokine secretion (Fig E2, A and B, in the Online Repository at www.jacionline.org). By single-vesicle flow cytometry, mGFP^+^ T-cell EV secretion did not differ between rested and activated T_H_2 cells from *Lck-Cre mTmG* mice until 24 hours of culture (Fig 2, B; Fig E2, C and D), at which point activated T_H_2 cells showed increased cell death (Fig 2, C; Fig E3, A, in the Online Repository at www.jacionline.org). Increased EV secretion by activated T_H_2 cells at 24 hours may be due to increased debris associated with cell death; thus, we used a 6-hour activation time point for our studies. Nanoparticle tracking analysis confirmed there was no difference in the quantity of EVs secreted by rested and activated T_H_2 cells at 6 hours of culture (Fig 2, D). By nanoparticle tracking analysis and transmission electron microscopy, there were also no size or morphologic differences between rested and activated T_H_2 cell EVs (Fig E3, B–D).

Because a hallmark of type 2 airway inflammation is lung eosinophilia, we investigated the effects of T_H_2 cell EVs on eosinophils. After EVs were purified by SEC from rested and activated T_H_2 cell culture media, mouse eosinophils differentiated from bone marrow (Fig E4, A–D, in the Online Repository at www.jacionline.org) were treated with 5 × 10^9^ EVs/mL, and viability was assessed with a membrane permeable dye (Fig 2, E). Treatment with rested EVs did not change survival from the nontreated condition; however, treatment with activated T_H_2 cell EVs increased eosinophil survival 2.5 times, equivalent to the survival benefit conferred by exogenous dosing of the canonical pro-survival cytokine IL-5 (Fig 2, F and G). Activated T_H_2 cell EVs promoted eosinophil survival through the inhibition of apoptosis, with decreased levels of annexin V binding and decreased caspase 3 activity in treated eosinophils (Fig 2, H–J). This effect on viability was dose dependent, with a half maximal effective concentration of 1.2 × 10^9^ EVs/mL (Fig 2, K), which was similar to the estimated physiologic concentration of T-cell EVs in airway lining fluid based on our genetic tracing studies from OVA and HDM models (2 × 10^9^ EVs/mL) (Fig E4, E). No effects of rested T_H_2 cell EVs were seen after treating eosinophils with increasing doses, suggesting a qualitative rather than quantitative difference in EVs secreted following T_H_2 cell activation through the TCR.

### T_H_2 cell EVs prolong eosinophilia and promote eosinophil survival *in vivo*

**FIG 3.**
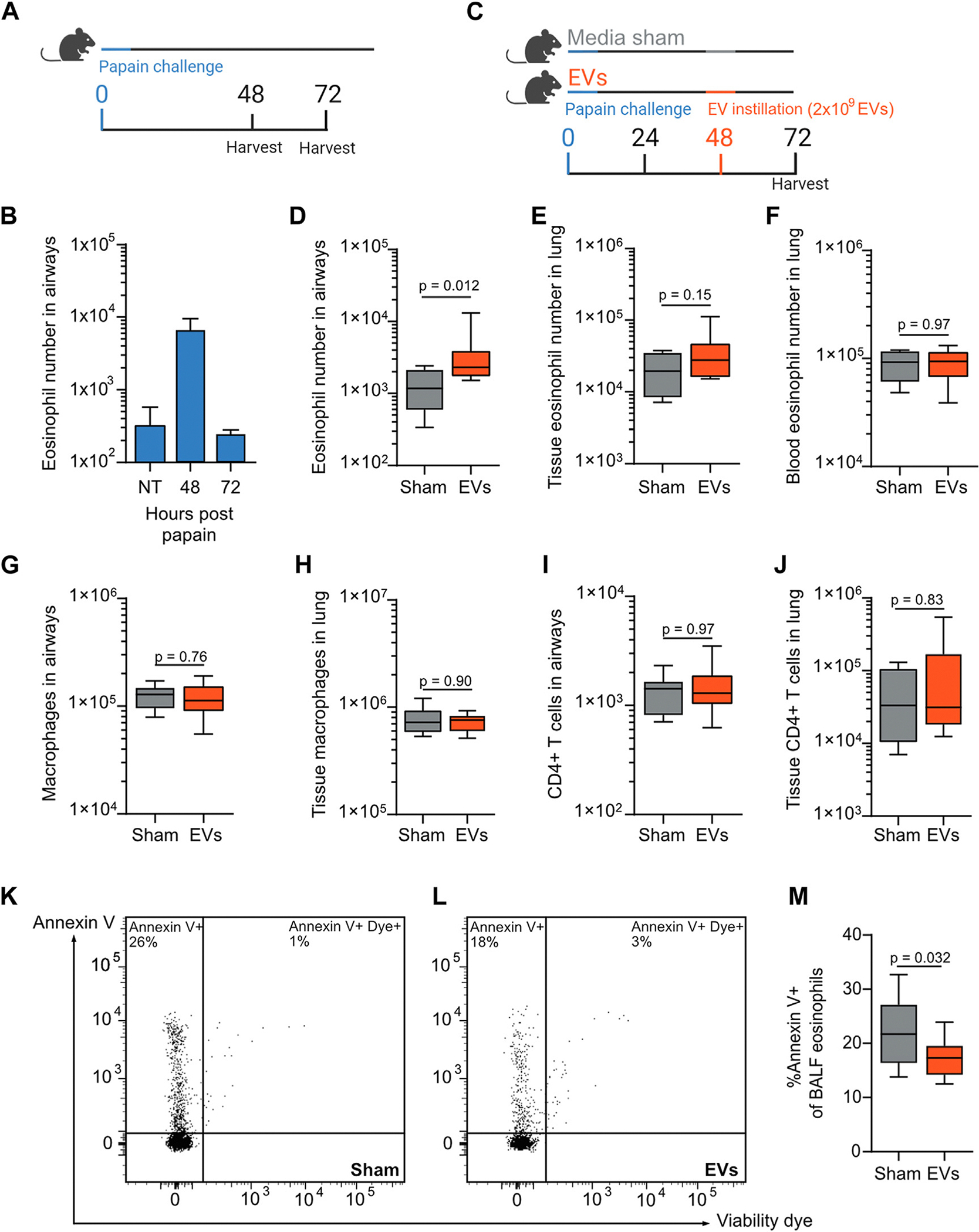
T_H_2 cell EVs prolong allergic eosinophilia and eosinophil survival *in vivo*. **(A)** Graphical depiction of papain airway challenge and BALF collection along a time course to assess eosinophil kinetics after aspiration of a single dose of 3.5 mg papain. **(B)** Quantification of BALF eosinophils in mice not treated with papain and at 48 and 72 hours following papain challenge. n = 4. **(C)** Graphical depiction of papain challenge and activated T_H_2 cell EV or sham control administration. **(D-F)** Quantification of *(D)* BALF eosinophils, *(E)* lung tissue eosinophils, and *(F)* blood eosinophils in the lungs of sham-treated and T_H_2 cell EV–treated mice at 72 hours following papain challenge. **(G** and **H)** Quantification of macrophages in the *(G)* BALF and *(H)* lungs of sham-treated and T_H_2 cell EV–treated mice at 72 hours following papain challenge. **(I** and **J)** Quantification of CD4^+^ T cells in the *(I)* BALF and *(J)* lungs of sham-treated and T_H_2 cell EV–treated mice at 72 hours following papain challenge. For *(D-J)*, n = 8 sham, n = 11 EV treated, 2 independent experiments, 2-tailed Mann-Whitney *U* test. **(K** and **L)** Representative flow cytometry plots showing % annexin V^+^ viability dye^−^ cells of eosinophils in BALF of *(K)* sham-treated and *(L)* T_H_2 cell EV–treated mice at 72 hours following papain challenge. **(M)** Quantification of annexin V^+^ viability dye^−^ eosinophils in the BALF of sham-treated and T_H_2 cell EV–treated mice at 72 hours following papain challenge. n = 11 sham, n = 11 EV treated, 2 independent experiments, 2-tailed Mann-Whitney *U* test. By flow cytometry, eosinophils were defined as SiglecF^+^ CD11b^+^ SSC^hi^, macrophages as F4/80^+^ CD11c^+^, and CD4^+^ T cells as TCRβ^+^ CD4^+^. *NT,* No treatment.

After establishing that activated T_H_2 cell EVs increase eosinophil viability *in vitro*, we hypothesized that activated T_H_2 cell EVs would prolong eosinophilia during airway inflammation *in vivo*. To minimize the contribution of endogenous T cell–derived EVs, we employed the papain model of airway inflammation, in which damage to the airway epithelium caused by protease activity of papain leads to the recruitment of eosinophils in the absence of an endogenous T-cell response.^31^ In our papain model, eosinophil numbers peaked at 48 hours following papain challenge and largely resolved by 72 hours (Fig 3, A and B). We found that administration of 2 × 10^9^ activated T_H_2 cell EVs at 48 hours after papain administration resulted in significantly increased SiglecF^+^CD11b^+^SSC^high^ eosinophils at 72 hours in the airways compared with mice treated with parallelly processed unconditioned media as a sham control (Fig 3, C and D). Interestingly, there was also a trend toward increased tissue eosinophils in the lung in EV-treated mice, whereas there was no difference in the number of eosinophils in the blood vessels in the lungs distinguished from tissue by intravascular anti-CD45 labeling (Fig 3, E and F). Total airway and lung tissue F4/80^+^CD11c^+^ macro-phages and TCRb^+^CD4^+^ T cells did not change with EV treatment, indicating a selective effect on eosinophils (Fig 3, G–J). Eosinophils from the BALF of EV-treated mice had reduced annexin V^+^ apoptotic eosinophils (Fig 3, K–M). Together, these data demonstrate that EVs produced from TCR-activated T_H_2 cells can promote eosinophil survival and prolong eosinophil-rich inflammation in the lung.

### T_H_2 cell EVs induce survival and activation pathways in eosinophils

**FIG 4.**
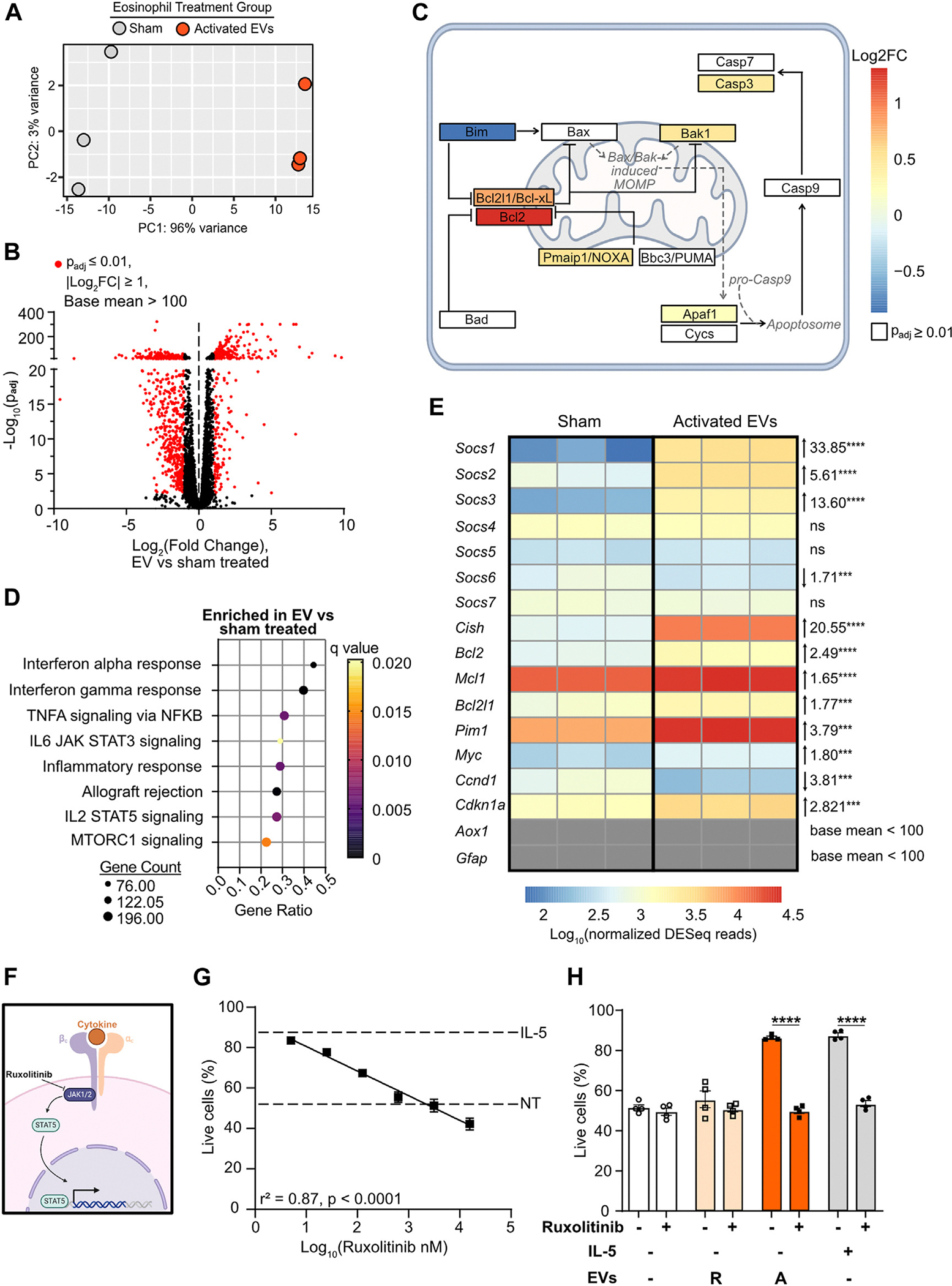
T_H_2 cell EVs induce survival and activation pathways in eosinophils. **(A)** Principal component analysis of gene expression differences between sham-treated eosinophils (n = 3) and activated T_H_2 cell EV–treated eosinophils (n = 3). **(B)** Volcano plot depicting differential gene expression between activated T_H_2 cell EV–treated and sham-treated eosinophils. Genes with adjusted *P* value ≤ .01 and |Log_2_FC| ≥1 are shown in *red*. **(C)** Diagram depicting Kyoto Encyclopedia of Genes and Genomes intrinsic apoptosis pathway genes, their relationships, and corresponding Log_2_ fold change in gene expression between activated T_H_2 cell EV–treated and sham-treated eosinophils. Genes that are upregulated in EV-treated eosinophils are shown in *boxes with warm colors*, genes that are downregulated in EV-treated eosinophils are shown in *boxes with cool colors*, and genes that are not differentially expressed are shown in *white boxes*. **(D)** Gene set enrichment analysis of activated T_H_2 cell EV–treated versus sham-treated eosinophils. Shown are gene sets with *q* value ≤ .05. **(E)** Heatmap depicting Kyoto Encyclopedia of Genes and Genomes JAK/STAT signaling pathway genes and corresponding normalized DESeq reads for those genes. Statistical analysis was performed using the R package DESeq2, and all *P* values are adjusted for multiple testing. **(F)** Diagram depicting JAK1/2 signaling downstream of a cytokine binding to its receptor. **(G)** Quantification of eosinophil survival following 24 hours of treatment with increasing doses of ruxolitinib (5, 25, 125, 625, 3,125, and 15,625 nM) and 5 × 10^9^ EVs/mL. n = 5, 2 independent experiments, simple linear regression. **(H)** Quantification of eosinophil survival following 24 hours of treatment with rested or activated T_H_2 cell EVs or IL-5 and 1.6 mM ruxolitinib. n = 4, 2-way ANOVA with Sidak correction for multiple comparisons. All error bars represent SEM, ***P < .001, ****P < .0001. *A,* Activated; *R,* rested.

To determine how T_H_2 cell EVs affect eosinophil viability, we assessed eosinophil gene expression by bulk RNA sequencing following treatment of cultured eosinophils with activated T_H_2 cell EVs or parallelly processed unconditioned media as a sham control. Principal component analysis showed that 96% of variance was due to EV treatment (Fig 4, A). In EV- versus sham-treated eosinophils, 369 genes were significantly (adjusted *P* < .01) upregulated (Log_2_ fold change ≥ 1), and 667 genes were significantly downregulated (Log_2_ fold change ≤ −1) (Fig 4, B; Tables E1 and E2 in the Online Repository at www.jacionline.org).

Consistent with protection of eosinophils from apoptosis with activated T_H_2 cell EV treatment (Fig 2, H–J), the intrinsic anti-apoptotic genes *Bcl2* and *Bcl2l1* (*Bcl-xL*) showed increased expression, and the intrinsic pro-apoptotic gene *Bcl2l11* (*Bim*) showed decreased expression in EV- versus sham-treated eosinophils (Fig 4, C). These genes are known to be regulated downstream of eosinophil survival pathways, including cytokine signaling, Fas/FasL signaling, CD40/CD40L signaling, TNF-*α*/fibronectin signaling, and Siglec-F cross-linking.^32–34^ We observed fewer significant changes in the extrinsic apoptosis pathway (Fig E5, A, in the Online Repository at www.jacionline.org).

Gene set enrichment analysis identified enrichment of gene sets involved in Janus kinase/signal transducer and activator of transcription (JAK/STAT)–dependent signaling in EV- versus sham-treated eosinophils (Fig 4, D). Consistent with this observation, we saw upregulation of many genes downstream of JAK/STAT signaling, including the pro-survival factors *Mcl1*, *Pim1*, and *Myc* and the negative regulators of cytokine signaling *Socs1*, *Socs2*, and *Socs3* (Fig 4, E). To test whether JAK/STAT signaling was required for eosinophil survival in response to activated T_H_2 cell EVs, we used the small-molecule JAK1/2 inhibitor ruxolitinib (Fig 4, F). Eosinophils cultured with either activated T_H_2 cell EVs or IL-5 demonstrated a dose-dependent inhibition of survival with ruxolitinib treatment (Fig 4, G; Fig E5, B and C). Treatment with ruxolitinib completely abrogated the survival benefit of activated T_H_2 cell EVs (Fig 4, H), indicating that activated T_H_2 cell EVs mediate eosinophil survival through JAK1/2 signaling. Additional gene sets enriched in EV- versus sham-treated eosinophils included inflammatory/immunity-related sets—TNF-*α*, IFN, inflammatory response, and allograft rejection (Fig 4, D). Several metabolism pathways were also regulated by EV treatment, with mTORC1 signaling genes increased and glycolysis genes decreased in EV-treated cells (Fig 4, D; Fig E5, D). Together, these data support that coordinated regulation of apoptosis in addition to inflammatory and metabolism pathways may allow eosinophils to both survive and participate in inflammatory responses after EV-mediated signaling from activated T_H_2 cells.

### IL-3 is a surface cargo carried on activated T_H_2 cell EVs

**FIG 5.**
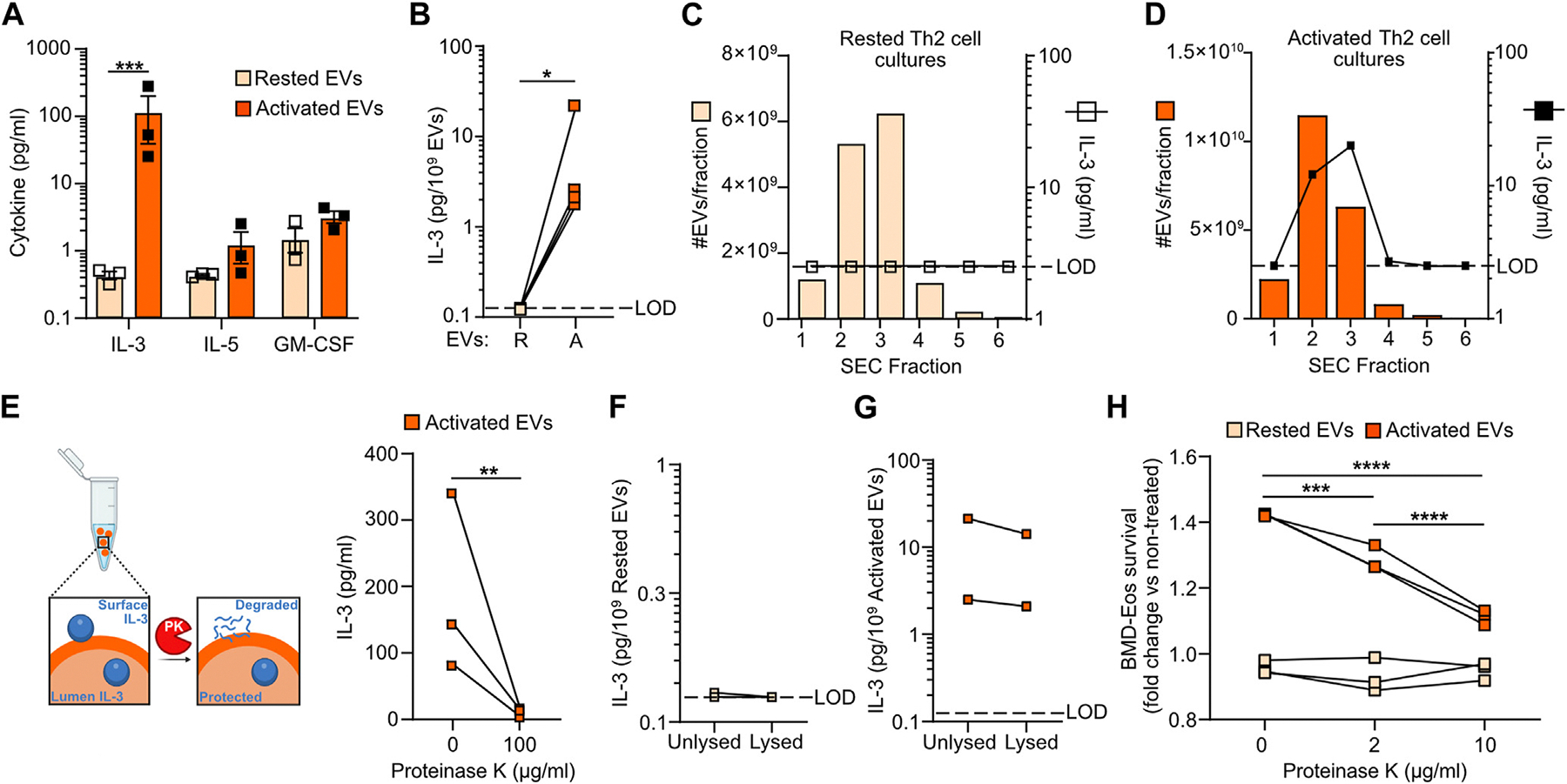
IL-3 is a surface cargo carried on activated T_H_2 cell EVs. **(A)** Quantification of the concentration of IL-3, IL-5, and GM-CSF in rested and activated T_H_2 cell EV SEC fractions as measured by cytokine array. n = 3, 2 independent experiments, 2-way ANOVA with Sidak correction for multiple comparisons. **(B)** Quantification of IL-3 present on SEC-purified rested and activated T_H_2 cell EVs measured by ELISA. n = 4, paired ratio 2-tailed *t* test. The limit of detection is defined by the manufacturer as 0.125 pg. **(C** and **D)** Quantification of EV number by nanoparticle tracking analysis *(left y-axis)* and IL-3 concentration by ELISA *(right y-axis)* in SEC fractions 1 to 6 collected from *(C)* rested and *(D)* activated T_H_2 cell culture media. The limit of detection is defined by the manufacturer as 2.5 pg/mL. **(E)** Graphical depiction *(left)* of proteinase K protection assay, in which purified activated T_H_2 cell EVs are incubated with 100 μg/mL proteinase K, proteinase K is removed by ultrafiltration, retained EVs are lysed, and IL-3 is detected by ELISA. Quantification *(right)* of IL-3 concentration by ELISA in activated T_H_2 cell EVs treated with and without 100 μg/mL proteinase K. n = 3, paired ratio 1-tail *t* test. **(F** and **G)** Quantification of EV-associated IL-3 by ELISA with and without lysis of *(F)* rested and *(G)* activated T_H_2 cell EVs purified from cell culture media by SEC and ultrafiltration. **(H)** Quantification of eosinophil survival following 24 hours of treatment with T_H_2 cell EVs subjected to prior proteinase K treatment compared with nontreated eosinophils. n = 3, 1-way ANOVA with Tukey multiple comparisons test. All error bars represent SEM, *P < .05, **P < .01, ***P < .001, ****P < .0001. *A,* Activated; *BMD,* bone marrow derived eosinophils; *LOD,* limit of detection; *R,* rested.

The common β chain family of cytokines signals through JAK/STAT and has critical roles in eosinophil survival and function.^35–37^ In our culture system, IL-3 was ≥40 times more abundant than the other β chain family members IL-5 and GM-CSF in preparations of purified EVs from T_H_2 cell cultures (Fig 5, A). Activated T_H_2 cell EVs carried an average of 6.9 pg IL-3 per 10^9^ EVs (minimum = 1.73, maximum = 21.3, SEM = 4.8) (Fig 5, B). The amount of EV-associated IL-3 significantly increased on activation through the TCR (Fig 5, A and B). Consistent with published data,^38^ T cells required TCR stimulation to produce large quantities of IL-3 (Fig E6, A and B, in the Online Repository at www.jacionline.org). In activated T_H_2 cell cultures, IL-3 was enriched in fractions containing the highest EV particle counts by nanoparticle tracking analysis (Fig 5, C and D). Cytokines can be carried both on the surface and within EVs.^19,28,39^ IL-3 was degraded by proteinase K in EV preparations, demonstrating that this cytokine is carried as a surface cargo (Fig 5, E). In further support of IL-3 being a surface cargo, equal amounts of IL-3 were detected by ELISA in lysed and unlysed samples (Fig 5, F and G). Finally, following surface shaving of EVs by proteinase K, activated T_H_2 cell EVs did not support eosinophil survival, indicating that a surface protein cargo was required for EV function (Fig 5, H; Fig E6, C–F).

### IL-3 cargo on T_H_2 cell EVs promotes eosinophil survival

**FIG 6.**
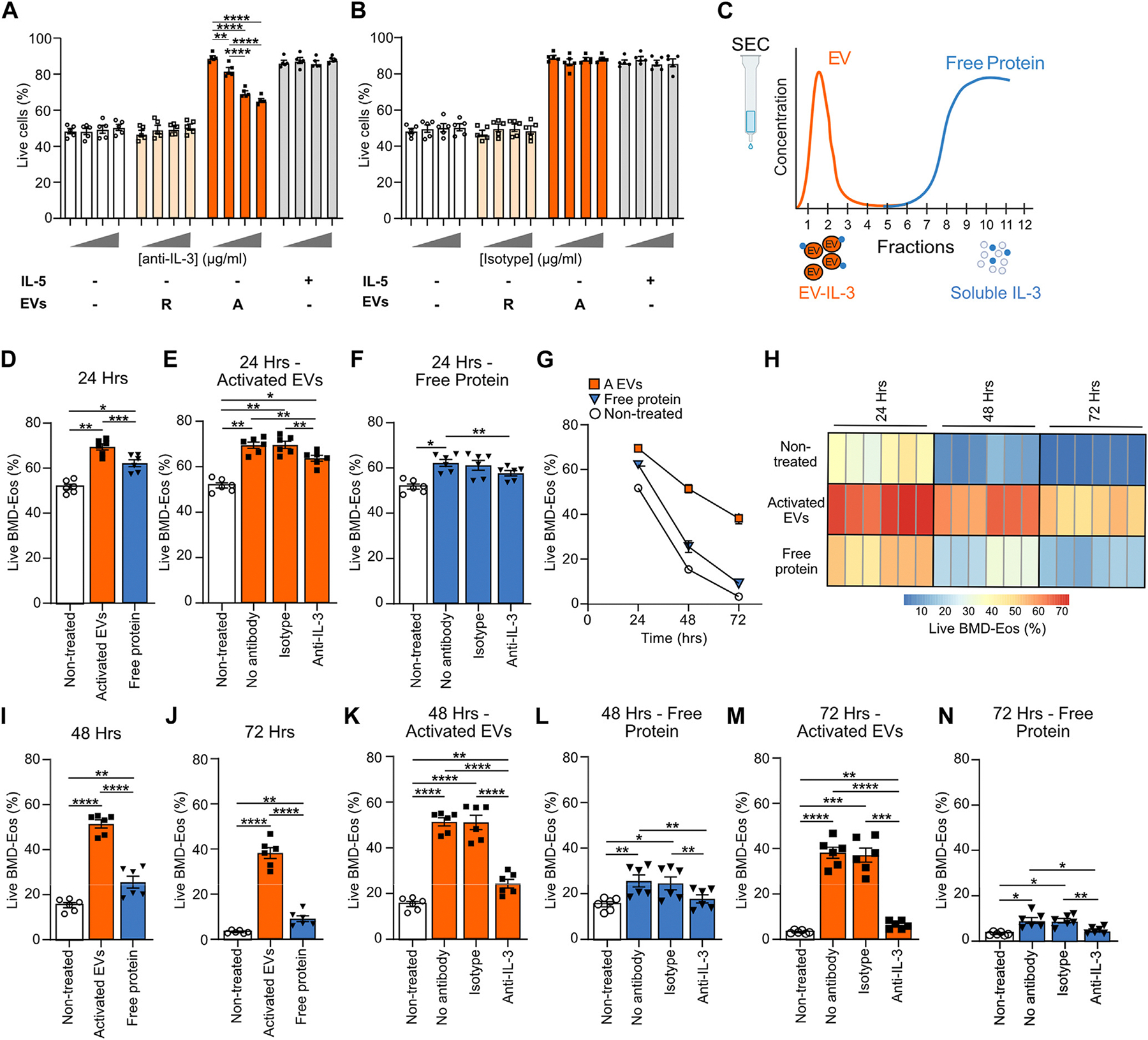
IL-3 cargo on T_H_2 cell EVs promotes eosinophil survival. (**A** and **B**) Quantification of eosinophil survival following 24 hours of treatment with rested or activated EVs pre-incubated with increasing concentrations of **(A)** anti-IL-3 or **(B)** isotype control (0, 1, 10, and 100 μg/mL). n = 5, 2-way ANOVA with Tukey correction for multiple comparisons, 2 independent experiments. **(C)** Graphical depiction of collection of EVs (fractions 1–4) and free proteins (fractions 9–12) by SEC. **(D)** Quantification of eosinophil survival following 24 hours of treatment with EV or free protein fractions normalized for IL-3 amount (2 pg/mL). **(E** and **F)** Quantification of eosinophil survival following 24 hours of treatment with EV or free protein and 100 μg/mL isotype control antibody or anti-IL-3. **(G)** Quantification of eosinophil survival over a time course (24, 28, and 72 hours) following treatment with EV or free protein fractions normalized for IL-3 amount (2 pg/mL). **(H)** Survival of individual eosinophil biological replicates averaged in *(G)* at 24, 48, and 72 hours. **(I** and **J)** Quantification of eosinophil survival following *(I)* 48 hours and *(J)* 72 hours treatment with EV or free protein fractions normalized for IL-3 amount (2 pg/mL). **(K** and **N)** Quantification of eosinophil survival following **(K** and **L)** 48 hours and **(M** and **N)** 72 hours treatment with EV or free protein and100 μg/mL isotype control antibody or anti-IL-3. For *(D-F)* and *(I-N)*, n = 6, 2 independent experiments, 1-way ANOVA with Tukey test for multiple comparisons. All error bars represent SEM, *P < .05, **P < .01, ***P < .001, ****P < .0001. *A,* Activated; *BMD,* bone marrow derived eosinophils; *R,* rested.

To determine whether EV-associated IL-3 is required for EVs to promote the survival of eosinophils, we performed antibody blocking experiments. Blocking of IL-3 significantly decreased the survival of eosinophils treated with activated T_H_2 cell EVs in a dose-dependent manner (Fig 6, A and B). Because cytokines can be both EV-associated and free proteins,^19,28,39^ we next compared the function of EV-containing (fractions 1–4) and free protein (fractions 9–12) preparations from T_H_2 cell cultures (Fig 6, C). We treated eosinophils with these fractions, equalizing for the amount of IL-3. Although both EVand free protein fractions supported eosinophil survival, the EV-containing fractions promoted a higher level of eosinophil survival than the free protein fractions at 24 hours (70% vs 62%, *P* = .0001) (Fig 6, D; Fig E7, A, in the Online Repository at www.jacionline.org). Antibody blocking experiments demonstrated that IL-3 contributed to the pro-survival effects of both sets of collected fractions (Fig 6, E and F). EVs with IL-3 were also highly effective at promoting eosinophil survival compared with recombinant cytokine (Fig E7, B and C). When cultures were examined over time, EVs were better able to sustain eosinophil survival than free protein fractions (51% vs 26% at 48 hours, *P* < .0001; 38% vs 9% at 72 hours, *P* < .0001) (Fig 6, G–J; Fig E7, D and E). Importantly, IL-3 was required for the pro-survival effects of activated T_H_2 cell EVs, even at these later time points (Fig 6, K–N). Together, these data support that EV-associated IL-3 from activated T_H_2 cells can serve as a communicating cargo that supports eosinophil survival.

## DISCUSSION

Therapies aimed at reducing eosinophil number through inhibiting IL-5/IL-5 receptor signaling are approved for clinical use in eosinophilic airway diseases, including asthma and chronic rhinosinusitis with nasal polyps.^40–47^ Identifying fundamental signaling pathways that promote eosinophilic inflammation allowed for the development of these novel and effective biological therapies. Continued investigations into basic mechanisms of eosinophil-rich type 2 inflammation will offer new therapeutic possibilities to care for patients with suboptimal or incomplete responses to therapy. Indeed, in at least some patients treated with anti-IL-5 therapy, eosinophils can remain in the lung and retain an activated phenotype, suggesting the presence of untargeted drivers of disease-causing eosinophilia.^48^ EVs have recently been identified as a form of cell-to-cell communication important for tissue inflammation.^49^ Although EV origins, numbers, and cargos are changed in respiratory biofluids during airway inflammation,^14,15,50–52^ how EVs communicate signals in the lung remain poorly understood.

In this study, we used membrane tracing strategies to identify that T-cell EVs are increased >10-fold in OVA and HDM models of type 2 airway inflammation. Furthermore, our results support that activated T_H_2 cell EVs are sufficient to promote eosinophil survival *in vitro* and *in vivo* and prolong eosinophilia in the lung through paracrine signaling. Our data also raise the possibility that EVs administered into the airspace may affect eosinophil numbers beyond the airway lumen in the lung parenchyma, which may be important for the therapeutic application of EVs. These findings together with our observations that T_H_2 cell EVs are sufficient to induce robust pro-survival, proinflammatory, and metabolic gene expression programs in eosinophils support the hypothesis that T_H_2 cell EVs participate as a signaling axis and a part of the unique local microenvironment in type 2 lung inflammation.

This work also highlights how changes in EV cargo are linked to cellular state and affect EV functions. Here, we found that although activation of T_H_2 cells through the TCR does not change the basic physical characteristics (ie, size, number, morphology) of secreted EVs, only EVs from reactivated T_H_2 cell EVs are capable of promoting eosinophil survival. These data suggest that polarized effector T cells in the tissues may need to encounter antigen to license EVs to promote inflammation. Future work is needed to test the function of EVs from other polarized effector cell populations (ie, T_H_1 and T_H_17) in airway inflammation, how cues from the environment may change EV secretion in allergen-driven airway inflammation, and whether the function of T-cell EVs changes in patients with asthma.

Although cytokines have largely been studied as freely soluble molecules, they have also recently been identified as surface and luminal EV cargos.^19,26,28,39^ In this study, we found that activated T_H_2 cell EVs carry the cytokine IL-3 as a surface cargo. Blocking this IL-3 dramatically reduced the ability of activated T_H_2 cell EVs to promote eosinophil survival. Although increases in IL-3 levels can be detected in patients with asthma and mouse models of allergic airway inflammation, and IL-3 can promote bone marrow production of eosinophils during inflammatory responses, including hypersensitivity reactions and parasite infections, the role of this cytokine in the pathogenesis of airway inflammatory diseases is poorly understood.^53–59^

Finally, a surprising finding was that T_H_2 cell EVs sustained eosinophil survival longer and more robustly than free protein fractions, despite dosing for equal IL-3 amounts. This observation suggests that EVs may potentiate cytokine function, possibly through targeting cytokines to recipient cells, protecting cytokine cargos from degradation, and/or changing signaling downstream of cytokine receptors. If EVs serve to enhance or amplify cytokine signaling in tissues, even low concentration EV-associated IL-3 may be a driver of lung inflammation. Eosinophilic inflammation is a hallmark of multiple diseases, including eosinophilic granulomatosis with polyangiitis, hypereosinophilic syndrome, and eosinophilic esophagitis.^60–62^ In addition, because cytokines mediate immune cell communication during virtually all inflammation-mediated pathologies, understanding the mechanisms by which EVs control cytokine function could lead to in-sights into how to treat both eosinophil-rich and other inflammatory diseases.

## Supplementary Material

1

2

3

4

## Key messages

- T cells secrete EVs in the airways in mouse models of allergic lung inflammation.
- Mouse T_H_2 cell EVs inhibit apoptosis and promote JAK/STAT pro-survival pathways in eosinophils.
- IL-3 carried on the surface of T_H_2 cell EVs is needed for this pro-survival effect, supporting a role for cytokine-carrying EVs as drivers of type 2 inflammation.

## Abbreviations used

BALF: Bronchoalveolar lavage fluid
EV: Extracellular vesicle
GFP: Green fluorescent protein
HDM: House dust mite
mGFP: Membrane-targeted green fluorescent protein
OVA: Ovalbumin
SEC: Size exclusion chromatography
TCR: T-cell receptor

## References

[1] Harker JA, Lloyd CM. T helper 2 cells in asthma. J Exp Med 2023;220:e20221094. 37163370 10.1084/jem.20221094 PMC10174188

[2] Hammad H, Lambrecht BN. The basic immunology of asthma. Cell 2021;184:1469–85. 33711259 10.1016/j.cell.2021.02.016

[3] McCracken JL, Tripple JW, Calhoun WJ. Biologic therapy in the management of asthma. Curr Opin Allergy Clin Immunol 2016;16:375–82. 27362324 10.1097/ACI.0000000000000284 PMC5405559

[4] van Niel G, D’Angelo G, Raposo G. Shedding light on the cell biology of extracellular vesicles. Nat Rev Mol Cell Biol 2018;19:213–28. 29339798 10.1038/nrm.2017.125

[5] Lee YJ, Kyeong JS, Young CC. Regulation of cargo selection in exosome biogenesis and its biomedical applications in cancer. Exp Mol Med 2024;56:877–89. 38580812 10.1038/s12276-024-01209-y PMC11059157

[6] Liu Y, Wang C. A review of the regulatory mechanisms of extracellular vesicles-mediated intercellular communication. Cell Commun Signal 2023;21:77. 37055761 10.1186/s12964-023-01103-6 PMC10100201

[7] Rodríguez M, Silva J, López-Alfonso A, López-Muñiz MB, Peña C, Domínguez G, Different exosome cargo from plasma/bronchoalveolar lavage in non-small-cell lung cancer. Genes Chromosomes Cancer 2014;53:713–24. 24764226 10.1002/gcc.22181

[8] Zareba L, Szymanski J, Homoncik Z, Czystowska-Kuzmicz M. EVs from BALF-mediators of inflammation and potential biomarkers in lung diseases. Int J Mol Sci 2021;22:3651. 33915715 10.3390/ijms22073651 PMC8036254

[9] Lal CV, Olave N, Travers C, Rezonzew G, Dolma K, Simpson A. Exosomal micro-RNA predicts and protects against severe bronchopulmonary dysplasia in premature infants. JCI Insight 2018;3:e93994. 29515035 10.1172/jci.insight.93994 PMC5922295

[10] Ransom MA, Bunn KE, Negretti NM, Jetter CS, Bressman ZJ, Sucre JMS, Developmental trajectory of extracellular vesicle characteristics from the lungs of preterm infants. Am J Physiol Lung Cell Mol Physiol 2023;324:L385–92. 36719083 10.1152/ajplung.00389.2022 PMC10026990

[11] Hough KP, Deshane JS. Exosomes in allergic airway disease. Curr Allergy Asthma Rep 2019;19:26. 30903454 10.1007/s11882-019-0857-3 PMC7117871

[12] Holtzman J, Heedoo L. Emerging role of extracellular vesicles in the respiratory system. Exp Mol Med 2020;52:887–95. 32541816 10.1038/s12276-020-0450-9 PMC7338515

[13] Takahashi T, Kato A, Berdnikovs S, Stevens WW, Suh LA, Norton JE, Microparticles in nasal lavage fluids in chronic rhinosinusitis: potential biomarkers for diagnosis of aspirin-exacerbated respiratory disease. J Allergy Clin Immunol 2017;140:720–9. 28238741 10.1016/j.jaci.2017.01.022 PMC5568994

[14] Hough KP, Landon SW, Trevor JL, Strenkowski JG, Maina N, Kim Y, Unique lipid signatures of extracellular vesicles from the airways of asthmatics. Sci Rep 2018;8:10340. 29985427 10.1038/s41598-018-28655-9 PMC6037776

[15] Pua HH, Happ HC, Gray CJ, Mar DJ, Chiou N, Hesse LE, Increased hematopoietic extracellular RNAs and vesicles in the lung during allergic airway responses. Cell Rep 2019;26:933–44. 30673615 10.1016/j.celrep.2019.01.002 PMC6365014

[16] Admyre C, Bohle B, Johansson SM, Focke-Tejkl M, Valenta R, Scheynius A, B cell-derived exosomes can present allergen peptides and activate allergen-specific T cells to proliferate and produce Th2-like cytokines. J Allergy Clin Immunol 2007;120:1418–24. 17868797 10.1016/j.jaci.2007.06.040

[17] Katz-Kiriakos E, Steinberg DF, Kluender CE, Osorio OA, Newsom-Stewart C, Baronia A, Epithelial IL-33 appropriates exosome trafficking for secretion in chronic airway disease. JCI Insight 2021;6:e136166. 33507882 10.1172/jci.insight.136166 PMC7934940

[18] Li F, Wang Y, Lin L, Wang J, Xiao H, Li J, Mast cell-derived exosomes promote Th2 cell differentiation via OX40L-OX40 ligation. J Immunol Res 2016;2016:3623898. 27066504 10.1155/2016/3623898 PMC4811108

[19] Fitzgerald W, Freeman ML, Lederman MM, Vasilieva E, Romero R, Margolis L. A system of cytokines encapsulated in extracellular vesicles. Sci Rep 2018;8:8973. Published correction appears in Sci Rep 2020;10:18935. 29895824 10.1038/s41598-018-27190-x PMC5997670

[20] Chiou N, Kageyama R, Ansel KM. Selective export into extracellular vesicles and function of tRNA fragments during T cell activation. Cell Rep 2018;25:3356–70. 30566862 10.1016/j.celrep.2018.11.073 PMC6392044

[21] Mittelbrunn M, Gutiérrez-Vázquez C, Villarroya-Beltri C, González S, Sánchez-Cabo F, González MÁ, Unidirectional transfer of microRNA-loaded exosomes from T cells to antigen-presenting cells. Nat Commun 2011;2:282. 21505438 10.1038/ncomms1285 PMC3104548

[22] Torralba D, Baixauli F, Villarroya-Beltri C, Fernández-Delgado I, Latorre-Pellicer A, Acín-Pérez R, Priming of dendritic cells by DNA-containing extracellular vesicles from activated T cells through antigen-driven contacts. Nat Commun 2018;9:2658. 29985392 10.1038/s41467-018-05077-9 PMC6037695

[23] Blanchard N, Lankar D, Faure F, Regnault A, Dumont C, Raposo G, TCR activation of human T cells induces the production of exosomes bearing the TCR/CD3/zeta complex. J Immunol 2002;168:3235–41. 11907077 10.4049/jimmunol.168.7.3235

[24] Torri A, Carpi D, Bulgheroni E, Crosti M, Moro M, Gruarin P, Extracellular microRNA signature of human helper T cell subsets in health and autoimmunity. J Biol Chem 2017;292:2903–15. 28077577 10.1074/jbc.M116.769893 PMC5314185

[25] Okoye IS, Coomes SM, Pelly VS, Czieso S, Papayannopoulos V, Tolmachova T, MicroRNA-containing T-regulatory-cell-derived exosomes suppress pathogenic T helper 1 cells. Immunity 2014;41:89–103. 25035954 10.1016/j.immuni.2014.05.019 PMC4104030

[26] Sullivan JA, Tomita Y, Jankowska-Gan E, Lema DA, Arvedson MP, Nair A, Treg-cell-derived IL-35-coated extracellular vesicles promote infectious tolerance. Cell Rep 2020;30:1039–51. 31995748 10.1016/j.celrep.2019.12.081 PMC7042971

[27] Martínez-Lorenzo MJ, Anel A, Gamen S, Monleón I, Lasierra P, Larrad L, Activated human T cells release bioactive Fas ligand and APO2 ligand in microvesicles. J Immunol 1999;163:1274–81. 10415024

[28] Hansen AS, Jensen LS, Gammelgaard KR, Ryttersgaard KG, Krapp C, Just J, T-cell derived extracellular vesicles prime macrophages for improved STING based cancer immunotherapy. J Extracell Vesicles 2023;12:e12350. Published correction appears in J Extracell Vesicles 2023;12:e12372. 37525396 10.1002/jev2.12350 PMC10390661

[29] Muzumdar MD, Tasic B, Miyamichi K, Li L, Luo L. A global double-fluorescent Cre reporter mouse. Genesis 2007;45:593–605. 17868096 10.1002/dvg.20335

[30] Chiang YJ, Hodes RJ. T-cell development is regulated by the coordinated function of proximal and distal Lck promoters active at different developmental stages. Eur J Immunol 2016;46:2401–8. 27469439 10.1002/eji.201646440 PMC5183457

[31] Halim TYF, Krauss RH, Sun AC, Takei F. Lung natural helper cells are a critical source of Th2 cell-type cytokines in protease allergen-induced airway inflammation. Immunity 2012;36:451–63. 22425247 10.1016/j.immuni.2011.12.020

[32] Ilmarinen P, Moilanen E, Kankaanranta H. Regulation of spontaneous eosinophil apoptosis—a neglected area of importance. J Cell Death 2014;7:1–9. 25278781 10.4137/JCD.S13588 PMC4167313

[33] Bureau F, Seumois G, Jaspar F, Vanderplasschen A, Detry B, Pastoret P, CD40 engagement enhances eosinophil survival through induction of cellular inhibitor of apoptosis protein 2 expression: possible involvement in allergic inflammation. J Allergy Clin Immunol 2002;110:443–9. 12209092 10.1067/mai.2002.126781

[34] Johnston LK, Bryce PJ. Understanding interleukin 33 and its roles in eosinophil development. Front Med (Lausanne) 2017;4:51. 28512632 10.3389/fmed.2017.00051 PMC5411415

[35] Dougan M, Dranoff G, Dougan SK. GM-CSF, IL-3, and IL-5 family of cytokines: regulators of inflammation. Immunity 2019;50:796–811. 30995500 10.1016/j.immuni.2019.03.022 PMC12512237

[36] Hercus TR, Kan WLT, Broughton SE, Tvorogov D, Ramshaw HS, Sandow JJ, Role of the β common (βc) family of cytokines in health and disease. Cold Spring Harb Perspect Biol 2018;10:a028514. 28716883 10.1101/cshperspect.a028514 PMC5983187

[37] Shen Z, Malter JS. Determinants of eosinophil survival and apoptotic cell death. Apoptosis 2015;20:224–34. 25563855 10.1007/s10495-014-1072-2 PMC5798882

[38] Dotsika EN, Sanderson CJ. Interleukin-3 production as a sensitive measure of T-lymphocyte activation in the mouse. Immunology 1987;62:665–8. 3501400 PMC1454149

[39] Erickson HL, Taniguchi S, Raman A, Leitenberger JJ, Malhotra SV, Oshimori N. Cancer stem cells release interleukin-33 within large oncosomes to promote immunosuppressive differentiation of macrophage precursors. Immunity 2024;57:1908–22. 39079535 10.1016/j.immuni.2024.07.004 PMC11324407

[40] Brusselle GG, Koppelman GH. Biologic therapies for severe asthma. N Engl J Med 2022;386:157–71. 35020986 10.1056/NEJMra2032506

[41] Nair P, Pizzichini MMM, Kjarsgaard M, Inman MD, Efthimiadis A, Pizzichini E, Mepolizumab for prednisone-dependent asthma with sputum eosinophilia. N Engl J Med 2009;360:985–93. 19264687 10.1056/NEJMoa0805435

[42] Haldar P, Brightling CE, Hargadon B, Gupta G, Monteiro W, Sousa A, Mepolizumab and exacerbations of refractory eosinophilic asthma. N Engl J Med 2009;360:973–84. 19264686 10.1056/NEJMoa0808991 PMC3992367

[43] Castro M, Mathur S, Hargreave F, Boulet L, Xie F, Young J, Reslizumab for poorly controlled, eosinophilic asthma: a randomized, placebo-controlled study. Am J Respir Crit Care Med 2011;184:1125–32. 21852542 10.1164/rccm.201103-0396OC

[44] Castro M, Zangrilli J, Wechsler ME, Bateman ED, Brusselle GG, Bardin P, Reslizumab for inadequately controlled asthma with elevated blood eosinophil counts: results from two multicentre, parallel, double-blind, randomised, placebo-controlled, phase 3 trials. Lancet Respir Med 2015;3:355–66. 25736990 10.1016/S2213-2600(15)00042-9

[45] Bjermer L, Lemiere C, Maspero J, Weiss S, Zangrilli J, Germinaro M. Reslizumab for inadequately controlled asthma with elevated blood eosinophil levels: a randomized phase 3 study. Chest 2016;150:789–98. 27056586 10.1016/j.chest.2016.03.032

[46] Bleecker ER, FitzGerald JM, Chanez P, Papi A, Weinstein SF, Barker P, Efficacy and safety of benralizumab for patients with severe asthma uncontrolled with high-dosage inhaled corticosteroids and long-acting β_2_-agonists (SIROCCO): a randomised, multicentre, placebo-controlled phase 3 trial. Lancet 2016;388:2115–27. 27609408 10.1016/S0140-6736(16)31324-1

[47] Bachert C, Sousa AR, Han JK, Schlosser RJ, Sowerby LJ, Hopkins C, Mepolizumab for chronic rhinosinusitis with nasal polyps: treatment efficacy by comorbidity and blood eosinophil count. J Allergy Clin Immunol 2022;149:1711–21. 35007624 10.1016/j.jaci.2021.10.040

[48] Kelly EA, Esnault S, Liu LY, Evans MD, Johansson MW, Mathur S, Mepolizumab attenuates airway eosinophil numbers, but not their functional phenotype, in asthma. Am J Respir Crit Care Med 2017;196:1385–95. 28862877 10.1164/rccm.201611-2234OC PMC5736971

[49] Buzas E The roles of extracellular vesicles in the immune system. Nat Rev Immunol 2023;23:236–50. 35927511 10.1038/s41577-022-00763-8 PMC9361922

[50] Levänen B, Bhakta NR, Paredes PT, Barbeau R, Hiltbrunner S, Pollack JL, Altered microRNA profiles in bronchoalveolar lavage fluid exosomes in asthmatic patients. J Allergy Clin Immunol 2013;131:894–903. 23333113 10.1016/j.jaci.2012.11.039 PMC4013392

[51] Paredes PT, Esser J, Admyre C, Nord M, Rahman QK, Lukic A, Bronchoalveolar lavage fluid exosomes contribute to cytokine and leukotriene production in allergic asthma. Allergy 2012;67:911–9. 22620679 10.1111/j.1398-9995.2012.02835.x

[52] Zhang M, Yu Q, Tang W, Wu Y, Lv J, Shi G, Epithelial exosome contactin-1 promotes monocyte-derived dendritic cell-dominant T-cell responses in asthma. J Allergy Clin Immunol 2021;148:1545–58. 33957164 10.1016/j.jaci.2021.04.025

[53] Kölle J, Zimmermann T, Kiefer A, Rieker RJ, Xepapadaki P, Zundler S, Targeted deletion of interleukin-3 results in asthma exacerbations. iScience 2022;25:104440. 35707726 10.1016/j.isci.2022.104440 PMC9189047

[54] Robinson DS, Hamid Q, Ying S, Tsicopoulos A, Barkans J, Bentley AM, Predominant Th2-like bronchoalveolar T-lymphocyte population in atopic asthma. N Engl J Med 1992;326:298–304. 1530827 10.1056/NEJM199201303260504

[55] Krammer S, Yang Z, Zimmermann T, Xepapadaki P, Geppert CI, Papadopoulos NG, An immunoregulatory role of interleukin-3 in allergic asthma. Front Immunol 2022;13:821658. 35281014 10.3389/fimmu.2022.821658 PMC8904351

[56] Mach N, Lantz CS, Galli SJ, Mihm M, Small C, Granstein R, Involvement of interleukin-3 in delayed-type hypersensitivity. Blood 1998;91:778–83. 9446636

[57] Lantz CS, Boesiger J, Song CH, Mach N, Kobayashi T, Mulligan RC, Role for interleukin-3 in mast-cell and basophil development and in immunity to parasites. Nature 1998;392:90–3. 9510253 10.1038/32190

[58] Du T, Martin JG, Powell WS, Renzi PM. IL-3 does not affect the allergic airway responses and leukotriene production after allergen challenge in rats. Eur Respir J 1999;13:970–5. 10414391 10.1034/j.1399-3003.1999.13e07.x

[59] Rignault-Bricard R, Machavoine F, Mecheri S, Hermine O, Schneider E, Dy M, IL-3-producing basophils are required to exacerbate airway hyperresponsiveness in a murine inflammatory model. Allergy 2018;73:2342–51. 29777594 10.1111/all.13480

[60] White J, Dubey S. Eosinophilic granulomatosis with polyangiitis: a review. Autoimmun Rev 2023;22:103219. 36283646 10.1016/j.autrev.2022.103219

[61] Biedermann L, Straumann A. Mechanisms and clinical management of eosinophilic oesophagitis: an overview. Nat Rev Gastroenterol Hepatol 2023;20:101–19. 36253463 10.1038/s41575-022-00691-x

[62] Helbig G, Klion AD. Hypereosinophilic syndromes—an enigmatic group of disorders with an intriguing clinical spectrum and challenging treatment. Blood Rev 2021;49:100809. 33714638 10.1016/j.blre.2021.100809

