# Supplementary material for: Mouse TH2 cell extracellular vesicles promote eosinophil survival through the surface cytokine cargo IL-3
Source: J Allergy Clin Immunol. Author manuscript; Available in PMC 2026 Jun 30. (PMC13318181; doi:10.1016/j.jaci.2025.05.027)
Supplement: 1 [file NIHMS2177599-supplement-1.pdf]

Supplemental Figure 1

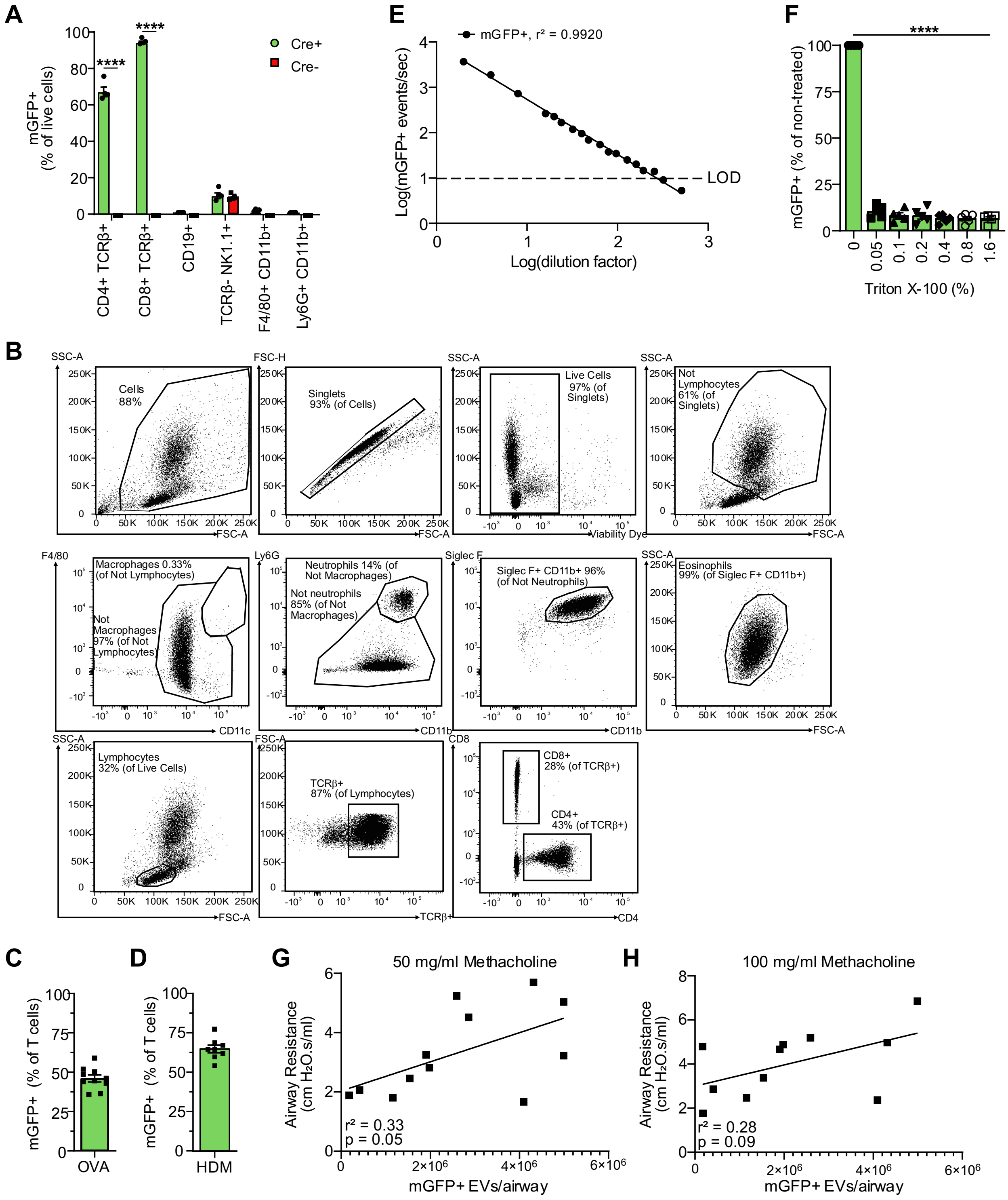

Supplemental Figure 1. BALF mGFP$^+$ EVs are detected in the linear range of single vesicle flow cytometry and are sensitive to Triton X-100 lysis. (A) Flow cytometric analysis of mGFP labeling of immune cell types in the spleens of Lck-Cre mTmG mice, including CD4+ T cells (CD4+TCRβ+), CD8+ T cells (CD8+TCRβ+), B cells (CD19+), natural killer cells (TCRβ-NK1.1+), macrophages/monocytes (F480+CD11b+), and neutrophils (Ly6G+CD11b+). n = 4, 2-way ANOVA with Tukey's test for multiple comparisons. (B) Representative flow cytometry gating scheme for quantification of macrophages, neutrophils, eosinophils, and T cells in the BALF in a mouse with airway inflammation. (C, D) Quantification of the percentage of mGFP+ T cells in the airways of (C) OVA and (D) HDM challenged mice, n = 10 OVA, n = 8 HDM, 2 independent experiments. (E) Flow cytometric analysis of mGFP+ event detection per second in BALF collected from a mouse with constitutive mGFP expression over a dilution series. n = 1, simple linear regression. (F) Quantification of mGFP+ particles detected in OVA Lck-Cre mTmG BALF following treatment with varying concentrations of TX-100. n = 5, one-way ANOVA (vs. 100%) with Bonferonni correction for multiple testing. (G, H) Correlation between airway resistance and the number of mGFP+ EVs in the BALF following induction of airway inflammation with HDM challenge at (G) 50 mg/ml and (H) 100 mg/ml methacholine. n = 12, simple linear regression. All error bars represent SEM, **** $p < 0.0001$.

Supplemental Figure 2

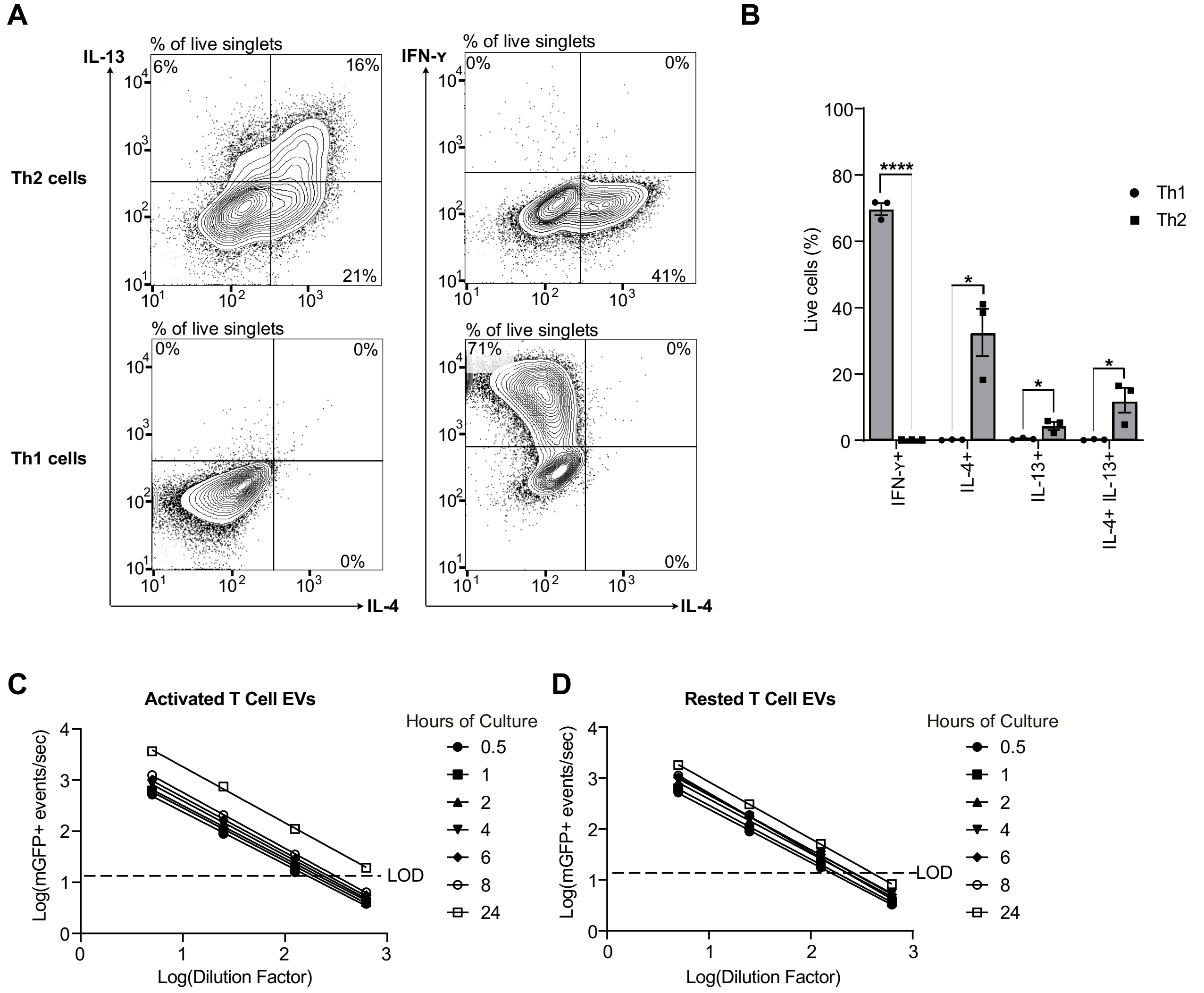

Supplemental Figure 2. T cells activate and polarize to Th2 cells in serum-free, chemically defined media. (A) Representative flow cytometry plots of IFNγ, IL-4, and IL-13 production by primary mouse $CD4^+$ T cells isolated from spleens and lymph nodes and then polarized with T cell receptor stimulation and either IL-4 and anti-IFNγ for Th2 cells (top) or IL-12 and anti-IL-4 for Th1 cells (bottom). **(B)** Quantification of IFNγ, IL-4, and IL-13 producing T cells following flow cytometric analysis. n = 3, 2-way ANOVA with Tukey's test for multiple comparisons. **(C, D)** Flow cytometric analysis of mGFP events detected per second in a dilution series that includes 5-, 25-, 125-, and 625-fold dilutions in media collected from **(C)** activated and **(D)** rested *Lck-Cre mTmG* T cells. n = 3, simple linear regression, r^2 all > 0.98. All errors bars represent SEM, * $p < 0.05$, **** $p < 0.0001$.

Supplemental Figure 3

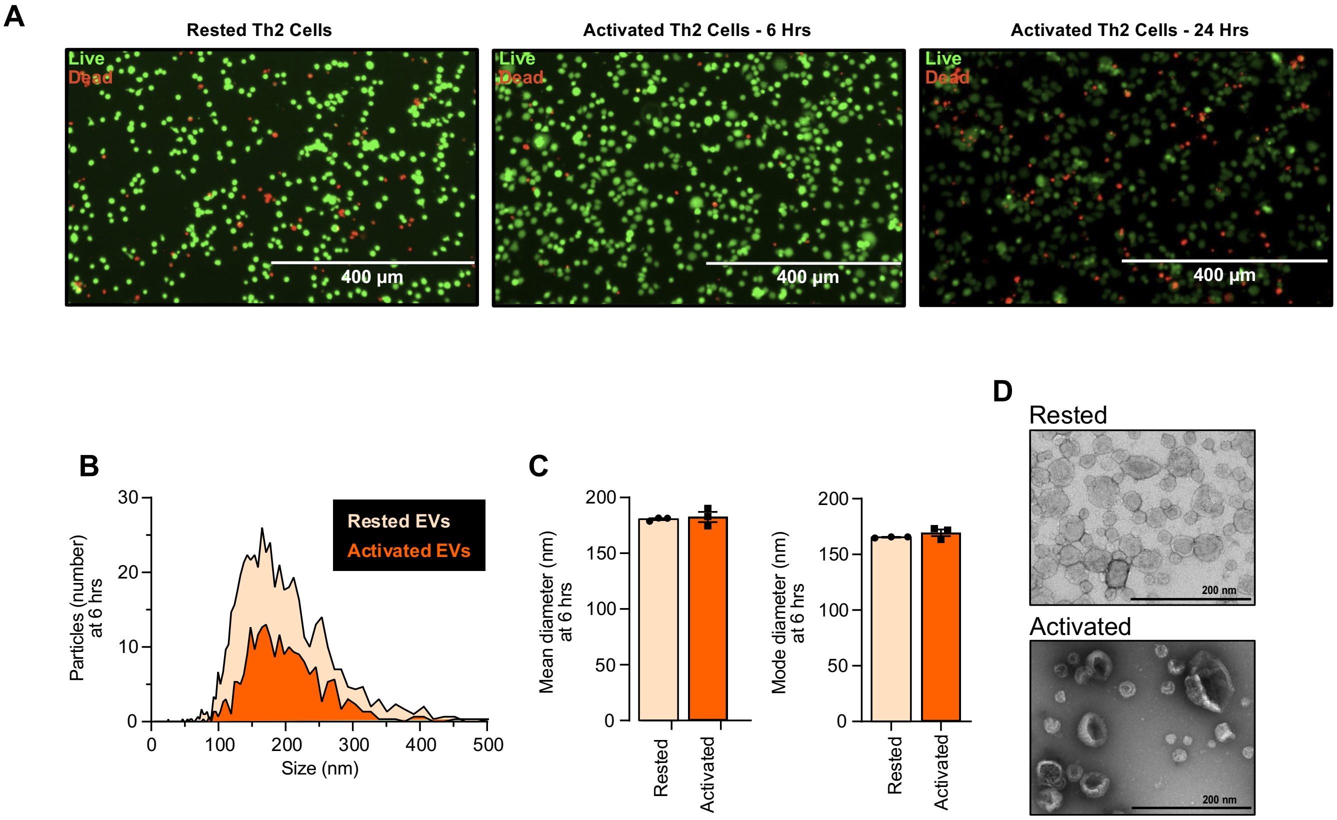

Supplemental Figure 3. Activation state does not change gross morphological characteristics of Th2 cell EVs. (A) Representative images of live/dead-stained rested (left), activated for 6 hours (middle), and activated for 24 hours (right) Th2 cells. **(B)** Average size distribution of EVs produced by rested (light orange) and activated (dark orange) Th2 cells at 6 hrs of culture as measured by nanoparticle tracking analysis. Histograms represent averages of 3 samples. **(C)** Mean and mode size of EVs produced by rested and activated Th2 cells at 6 hrs of culture as measured by nanoparticle tracking analysis. n =3, two-tailed t test. **(D)** Transmission electron micrographs of EVs purified by density gradient from rested (top) and activated (bottom) Th2 cell culture media, magnification 1100x. All error bars represent SEM.

Supplemental Figure 4

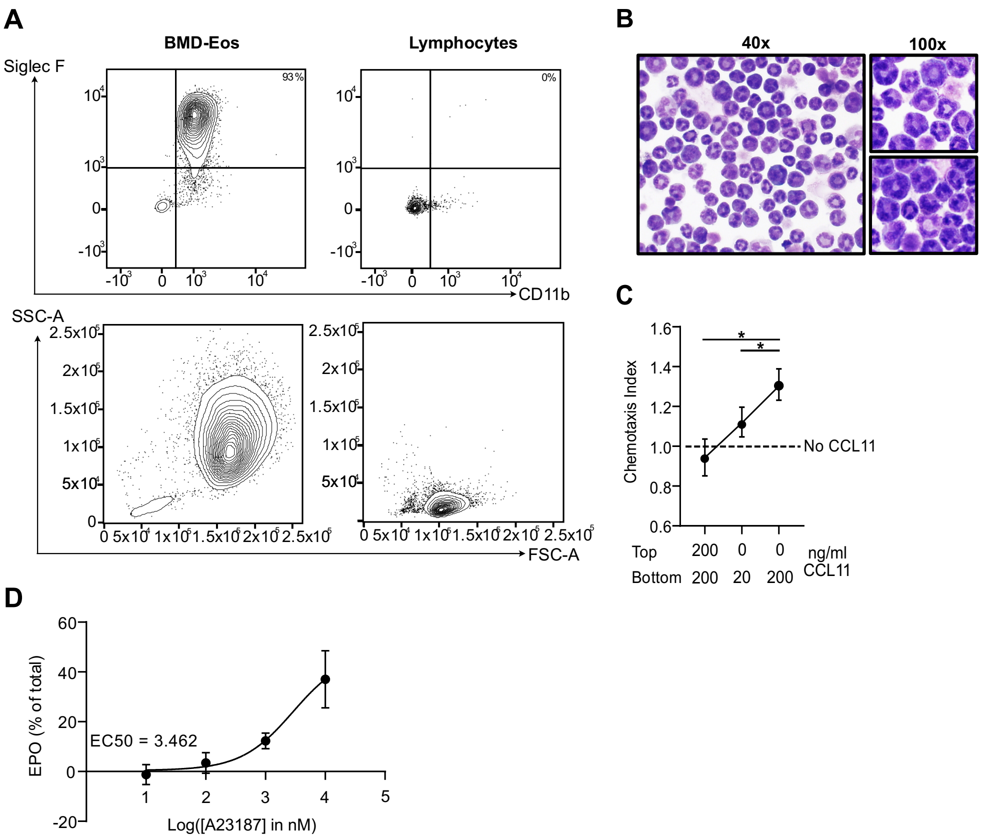

E

Estimated physiological concentration of T cell EVs in the airway lining fluid during mouse model of type 2 inflammation

4.62×10^6 mGFP+ T cell EVs (mean number of mGFP+ EVs in OVA BALF, see Fig. 1K) / 1 ml (volume of BALF wash) x 100 (dilution factor of BALF) x 5 (sensitivity correction for NPT vs EV flow cytometry, 200:40, see Fig. 2B & 2D) = 2.31×10^9 T cell EVs/ml

Supplemental Figure 4. Eosinophils differentiated from mouse bone marrow recapitulate eosinophil functions *in vitro*. (A) Representative flow cytometry plots of day 12 bone marrow-derived eosinophils (left) and lymphocytes (right) stained for the eosinophil markers Siglec F and CD11b (top) and assayed for forward- and side-scatter (bottom). **(B)** Light micrographs of day 12 bone marrow-derived eosinophils stained with hemacolor staining kit at 40x and 100x magnification. **(C)** Quantification of day 12 bone marrow-derived eosinophil chemotaxis to the eotaxin CCL11 by a transwell migration assay. n = 3, one-way ANOVA with Tukey's multiple comparisons test. **(D)** Quantification of day 12 bone marrow-derived eosinophil Eosinophil Peroxidase (EPO) production in response to increasing doses of the calcium ionophore A23187, n = 3, log[agonist] vs response with variable response (4 parameters). **(E)** Formula for calculating the estimated physiological concentration of T cell EVs in airway lining fluid during allergic inflammation. NPT = nanoparticle tracking. All error bars represent SEM, * $p < 0.05$.

Supplemental Figure 5

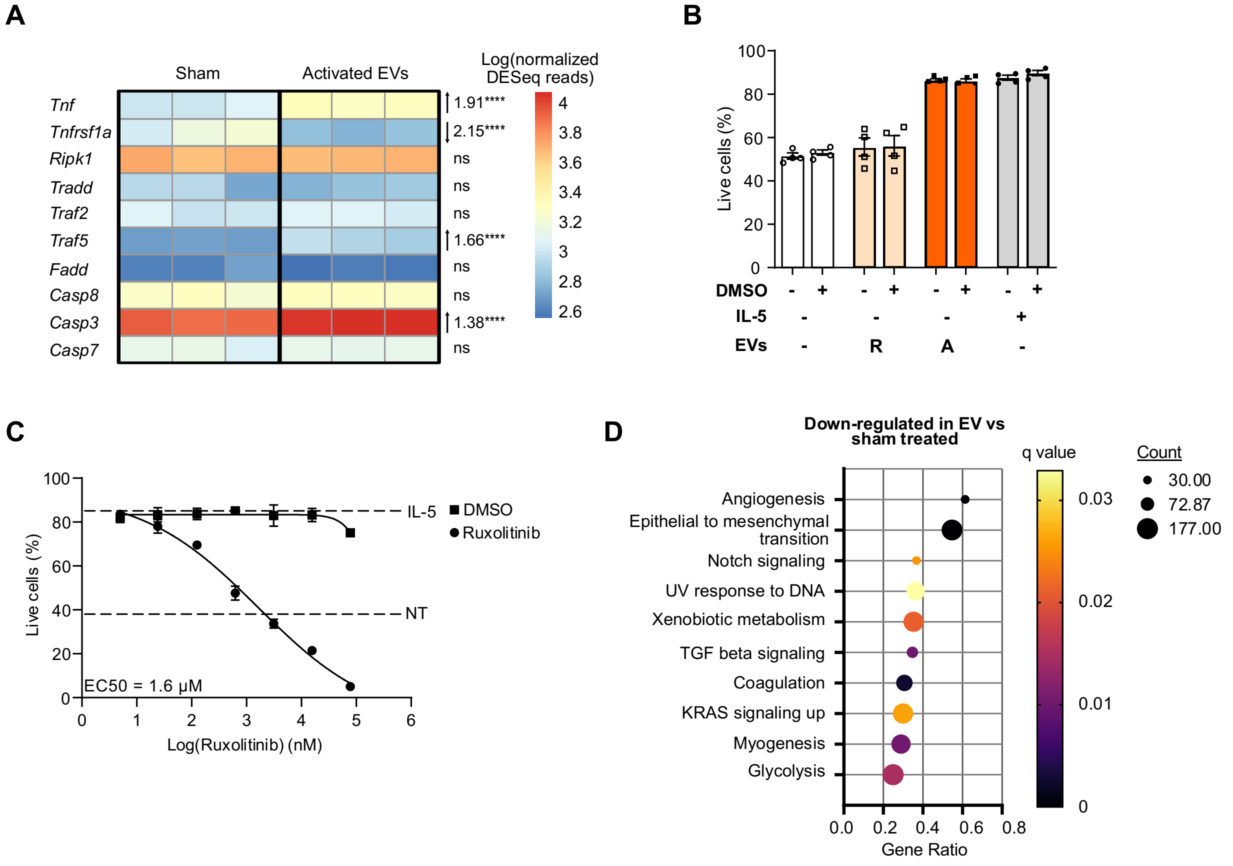

E

Sample	Total sequences	Sequences flagged as poor quality	Sequence length
**Sham 1**	86747212	0	151
**Sham 2**	68137746	0	151
**Sham 3**	64887648	0	151
**Activated EV 1**	87512214	0	151
**Activated EV 2**	102304965	0	151
**Activated EV 3**	94383144	0	151

Supplemental Figure 5. Activated T cell EVs regulate metabolism pathways and extrinsic apoptosis genes in eosinophils *in vitro*. (A) Heatmap depicting KEGG extrinsic apoptosis pathway genes and corresponding normalized DESeq reads for those genes in sham treated and activated Th2 cell EV treated bone marrow eosinophils. **(B)** Quantification of bone marrow derived eosinophil survival following 24 hrs treatment with rested or activated Th2 cell EVs or IL-5 with or without DMSO, n = 4, 2-way ANOVA with Sidak's correction for multiple comparisons. **(C)** Quantification of bone marrow derived eosinophil survival following 24 hrs treatment with increasing doses of ruxolitinib or vehicle (DMSO) and 10 ng/mL IL-5, n = 3, log[agonist] vs response with variable response (4 parameters). **(D)** Gene set enrichment analysis of activated Th2 cell EV treated vs sham treated bone marrow derived eosinophils. Shown are gene sets downregulated in EV treated vs sham treated cells with q value ≤0.05. Differential gene expression analysis was performed using the R package DESeq2. All error bars represent SEM, **** $p < 0.0001$. **(E)** Table depicting quality metrics of RNA-sequencing data generated by FastQC.

Supplemental Figure 6

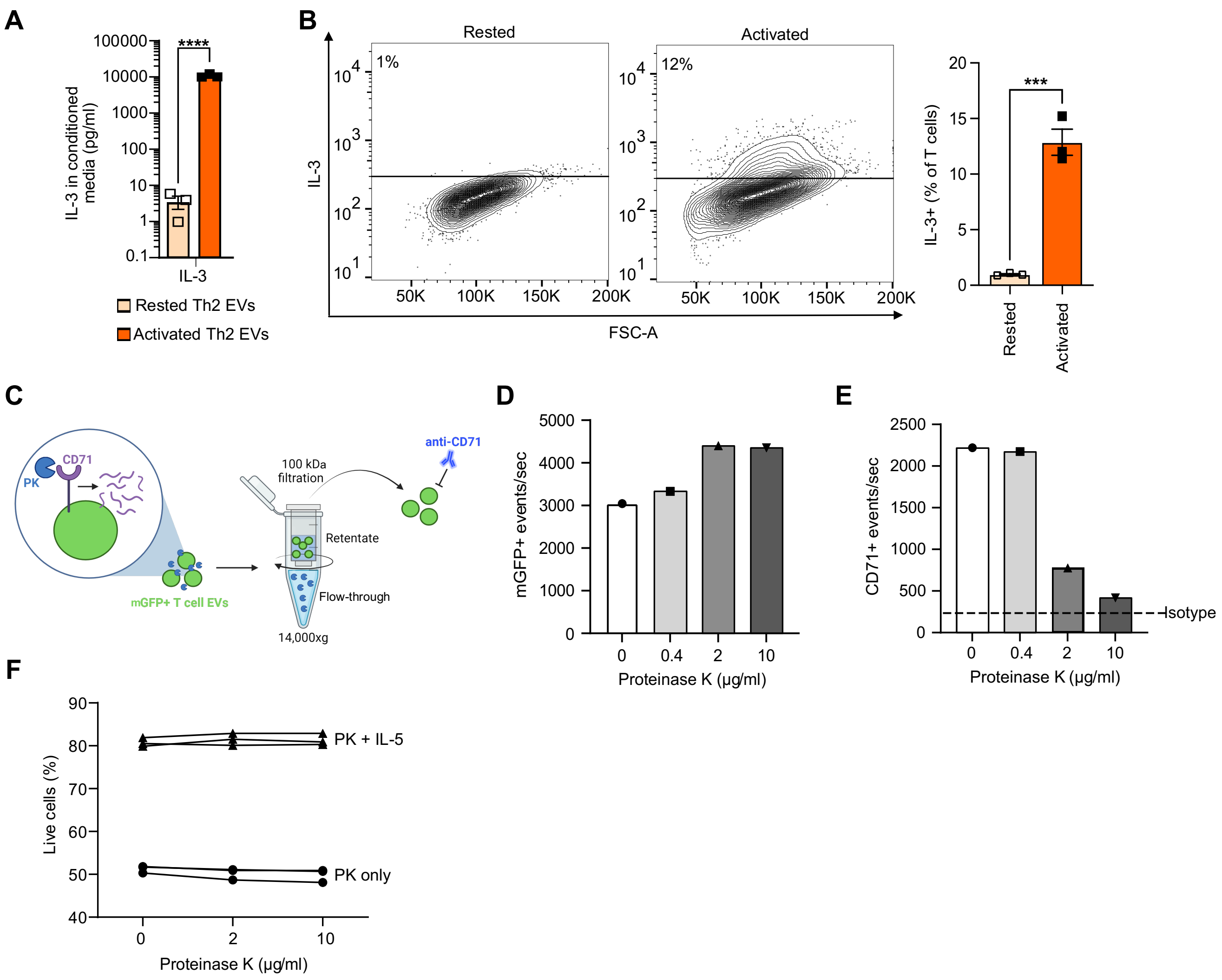

Supplemental Figure 6. IL-3 is on the surfaces of EVs secreted by activated Th2 cells. (A) Quantification of IL-3 in unfractionated conditioned media from rested and activated Th2 cell culture media. n = 3, 2 independent experiments, 2-way ANOVA with Sidak's test for multiple comparisons. **(B)** Representative flow cytometry plots (left) and quantification (right) of percent of IL-3+ Th2 cells in rested conditions and after re-stimulation through the T cell receptor for 6 hrs. n = 3, one-tailed t test. **(C)** Because eosinophil viability is sensitive to proteinase K (PK) treatment, EVs must be separated from PK before they are added to cell culture. Graphical depiction of EV PK treatment purification and validation strategy. mGFP^{+} T cell EVs were incubated with PK, after which they were subjected to centrifugal filtration using a 100 kDa pore size. The flow-through containing PK was discarded, and the retentate containing EVs was stained with a fluorescent antibody against CD71, an abundant T cell EV surface cargo. EVs were then subjected to single vesicle flow cytometry to assess the integrity of the EVs and confirm surface protein cargo degradation. **(D)** Quantification of the number of mGFP^{+} events detected per second in the retentate in *Lck-Cre mTmG* T cell culture media after treatment with increasing concentrations of PK. **(E)** Quantification of the number of CD71^{+} events detected per second in the retentate in *Lck-Cre mTmG* T cell culture media after treatment with increasing concentrations of PK. For **(D)** and **(E)**, n = 1 set of T cell EVs pooled from 3 mice. The dotted line depicts fluorescent events detected per second with an isotype control antibody. **(F)** Quantification of cell death following bone marrow derived eosinophil treatment with sham PK control retentate with or without IL-5. Unconditioned cell culture media parallelly processed as EV-containing media was treated with PK as depicted in **(C)**. n = 3, 2-way ANOVA with Tukey's multiple comparison test (comparison of PK doses within PK only or PK + IL-5 conditions).

Supplemental Figure 7

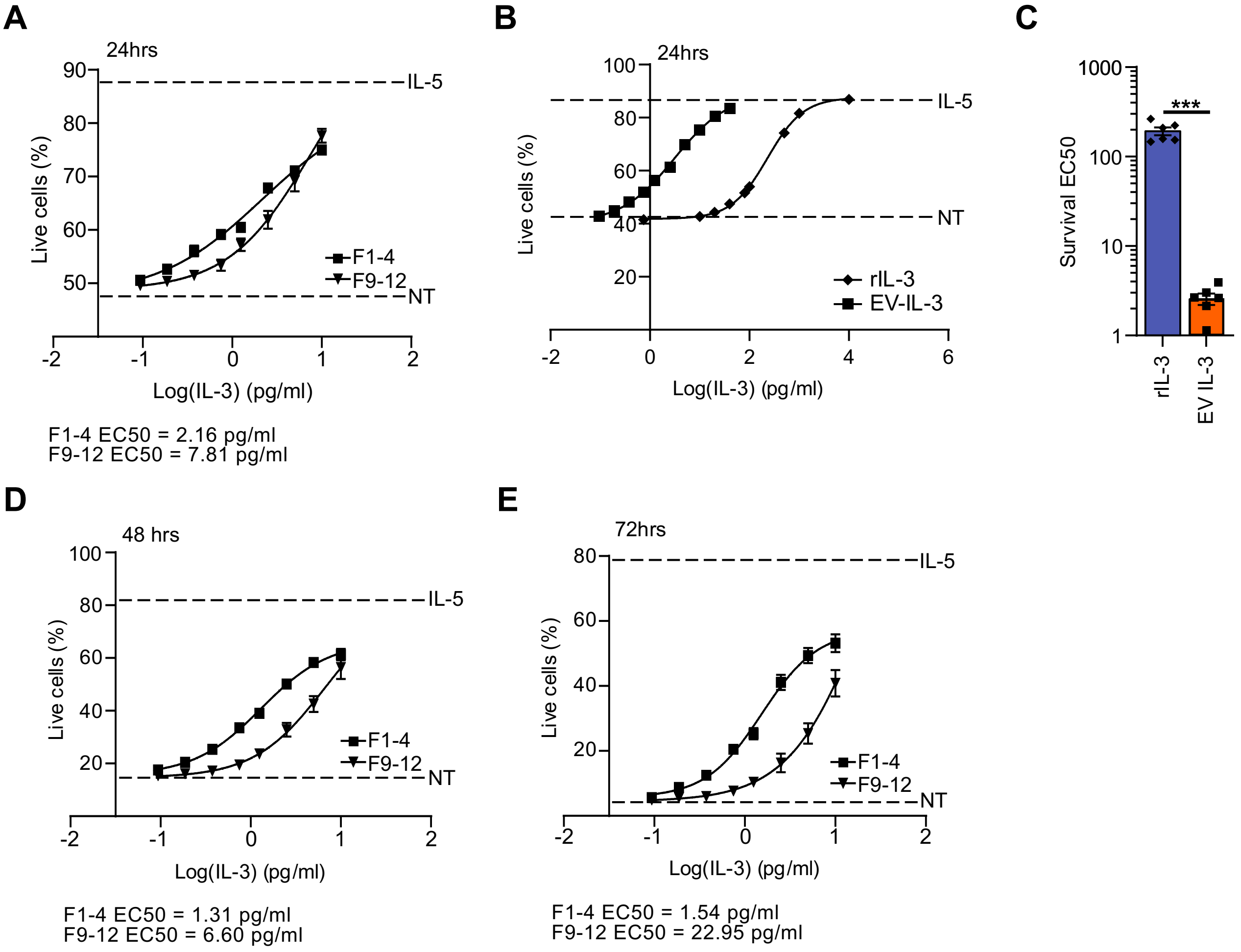

Supplemental Figure 7. EV-associated IL-3 dose curves compared to free protein fractions and recombinant IL-3. (A) Quantification of bone marrow derived eosinophil survival following 24 hrs of treatment with increasing doses of activated Th2 cell EV-containing fractions (1-4) or free protein fractions (9-12). Plotted is survival against amount of IL-3 in those fractions quantified by ELISA (0.09375, 0.1875, 0.375, 1.25, 2.5, 5, 10 pg/ml), n = 3, log[agonist] vs response with variable slope (4 parameters), representative of 2 independent experiments. **(B)** Quantification of eosinophil survival following 24 hrs treatment with increasing doses of activated Th2 cell EV with IL-3 quantified by ELISA (0.09375 pg/mL, 0.1875 pg/mL, 0.375 pg/mL, 0.75 pg/mL, 1.25 pg/mL, 2.5 pg/mL, 5 pg/mL, 10 pg/mL, 20 pg/mL, and 40 pg/mL) or soluble recombinant IL-3 (0.75 pg/mL, 1.25 pg/mL, 2.5 pg/mL, 5 pg/mL, 10 pg/mL, 20 pg/mL, 40 pg/mL, 80 pg/mL, 100 pg/mL, 500 pg/mL, 1000 pg/mL, and 10,000 pg/mL), n = 3, log[agonist] vs response with variable slope (4 parameters), representative of 2 independent experiments. **(C)** Comparison of eosinophil survival EC50 of soluble recombinant IL-3 or activated Th2 cell EV-associated IL-3, n = 6, 2 independent experiments, paired two-tailed t test. **(D,E)** Quantification of eosinophil survival following **(D)** 48 hrs or **(E)** 72 hrs of treatment with increasing doses of activated Th2 cell EV-containing fractions (1-4) or free protein fractions (9-12). Plotted is survival against amount of IL-3 quantified by ELISA (0.09375, 0.1875, 0.375, 1.25, 2.5, 5, 10 pg/ml), n = 3, log[agonist] vs response with variable slope (4 parameters), representative of 2 independent experiments. *** $p < 0.001$.
